# TCRβ-expressing macrophages induced by a pathogenic murine malaria correlate with parasite burden and enhanced phagocytic activity

**DOI:** 10.1371/journal.pone.0201043

**Published:** 2018-07-25

**Authors:** Miranda S. Oakley, Joanna K. Chorazeczewski, Maya Aleshnick, Vivek Anantharaman, Victoria Majam, Bhavna Chawla, Timothy G. Myers, Qin Su, Winter A. Okoth, Kazuyo Takeda, Adovi Akue, Mark KuKuruga, L. Aravind, Sanjai Kumar

**Affiliations:** 1 Division of Bacterial, Parasitic, and Allergenic Products, Office of Vaccines Research and Review, Center for Biologics Evaluation and Research, Food and Drug Administration, Silver Spring, MD, United States of America; 2 Division of Emerging and Transfusion Transmitted Diseases, Office of Blood Research and Review, Center for Biologics Evaluation and Research, Food and Drug Administration, Silver Spring, MD, United States of America; 3 National Center for Biotechnology Information, National Library of Medicine, NIH, Bethesda, MD, United States of America; 4 Genomics Technologies Section, Research Technologies Branch, National Institute of Allergy and Infectious Diseases, NIH, Bethesda, MD, United States of America; University of Michigan Health System, UNITED STATES

## Abstract

Macrophages express a wide array of invariant receptors that facilitate host defense and mediate pathogenesis during pathogen invasion. We report on a novel population of CD11b^high^CD14^+^F4/80^+^ macrophages that express TCRβ. This population expands dramatically during a *Plasmodium berghei* ANKA infection and sequesters in the brain during experimental cerebral malaria. Importantly, measurement of TCRβ transcript and protein levels in macrophages in wildtype versus nude and *Rag1* knockout mice establishes that the observed expression is not a consequence of passive receptor expression due to phagocytosis or trogocytosis of peripheral T cells or nonspecific antibody staining to an Fc receptor or cross reactive epitope. We also demonstrate that TCRβ on brain sequestered macrophages undergoes productive gene rearrangements and shows preferential Vβ usage. Remarkably, there is a significant correlation in the proportion of macrophages that express TCRβ and peripheral parasitemia. In addition, presence of TCRβ on the macrophage also correlates with a significant increase (1.9 fold) in the phagocytosis of parasitized erythrocytes. By transcriptional profiling, we identify a novel set of genes and pathways that associate with TCRβ expression by the macrophage. Expansion of TCRβ-expressing macrophages points towards a convergence of the innate and adaptive immune responses where both arms of the immune system cooperate to modulate the host response to malaria and possibly other infections.

## Introduction

Depending on the *Plasmodium* species and the immune status of the host, infection with malaria parasites may be asymptomatic and mild or acute and fulminant which can result in death. Severe malaria anemia (SMA) and cerebral malaria (CM) are the two major clinical syndromes which are associated with fatalities caused by malaria infection [1]. Macrophages, a component of the innate immune system, play both protective and pathogenic roles during malaria infection. The protective function of macrophages is mediated through a receptor-dependent phagocytic clearance of infected erythrocytes in the spleen [2, 3], or an antibody-dependent cellular mechanism that engages the Fcγ receptors [4, 5].

Several lines of evidence suggest that parasite burden alone cannot account for the level of SMA observed during acute and chronic malaria and this phenomenon is mediated by multiple host factors [6, 7]. Macrophages may contribute towards SMA through several independent mechanisms including removal of both infected and uninfected erythrocytes [8, 9] and/or by influencing the generation of new erythrocytes through suppression of erythropoiesis [7, 10] and increased dyserythropoiesis [11]. Deformity of erythrocytes [12], altered expression of complement regulatory proteins, and enhanced opsonin deposition [13] [14] during malaria infection render infected and uninfected erythrocytes susceptible to phagocytic clearance by macrophages.

Macrophages also play a pathogenic role in cerebral malaria. *P*. *berghei* ANKA (*Pb−A*), a highly virulent mouse malaria, is used to study experimental cerebral malaria (ECM) in susceptible strains of mice [15]. A hallmark of ECM caused by a *Pb−A* infection in C57BL/6 mice is the sequestration of brain infiltrating monocytes/macrophages; early but not late depletion of monocytes/macrophages with a liposome containing dichloromethylene diphosphate prevents the development of ECM [16, 17]. Furthermore, accumulation of monocytes with phagocytosed hemozoin within the brain microvessels has been documented in Malawian children with CM [18]. Moreover, autopsy confirmed cases of fatal pediatric CM have significantly more (greater than 600 times) brain intravascular monocytes than children with other causes of death [19].

Immune cells of both myeloid and lymphoid origins utilize surface and cytosolic receptors to perform their defense and other immunological functions. Conventionally, myeloid receptors are invariant while lymphoid cells utilize combinatorial variant receptors.

Although the presence of variant immunoreceptors on macrophages is unconventional, recent studies have reported TCR expression by non-lymphoid cells [20], including neutrophils [21] [22], eosinophils [23], and macrophages. TCRβ-expressing macrophages have recently been identified in tuberculosis granulomas [24], atherosclerotic lesions [25], and the tumor microenvironment [26]. Beham *et al*. reported that 87% of macrophages located in the innermost segment of the epithelioid cell corona of human caseous granulomas expressed TNF*−*α inducible TCRαβ. In this tuberculosis study, stimulation of macrophage TCR enhanced phagocytosis and increased production of proinflammatory cytokines such as CCL2. Similarly, Fuchs *et al*. showed that >80% of lesion macrophages express cholesterol-responsive TCRαβ in advanced human carotid artery atherosclerosis. Furthermore, in several types of cancer, > 40% of the tumor macrophage population expressed TCRαβ in randomly selected human tumor entities (colon cancer, esophageal cancer, hepatic carcinoma, and melanoma). In summary, these studies show that high numbers of TCRαβ-expressing macrophages accumulate at the site of pathogenesis in three types of disease.

In this study, we identified a novel population of CD11b^high^CD14^+^F4/80^+^ macrophages that express combinatorial TCRβ during a *Pb−A* infection. Importantly, measurement of TCRβ transcript and protein levels of macrophages in wildtype (WT) versus nude and *rag1* knockout (KO) mice confirms that TCRβ expression by the macrophage is not an artifact of 1) nonspecific anti-TCRβ binding to a cross-reactive epitope or Fc receptor on the macrophage surface or 2) passive receptor expression due to phagocytosis or trogocytosis (membrane swapping) of peripheral T cells. Further studies will be needed to discern the function of combinatorial TCRβ receptors on macrophages in malaria, their role in immunity and pathogenesis and the parasite moieties that stimulate their expansion and migration to the brain.

## Material and methods

### Ethics statement

All mice were maintained at the Food and Drug Administration animal care facility and treated according to the guidelines set by the Animal Care and Use Committee. All animal experiments were conducted under a protocol (ASP 2009–22) that was approved by the Animal Care and Use Committee of CBER, FDA.

### Mice and parasite infections

Six to ten week old female C57BL/6, Balb/c, B6.129S7*−Rag1*^*tm1Mom*^/J, and B6.Cg*−Foxn1*^*nu*^/J (nude) mice were purchased from The Jackson Laboratory (Bar Harbor, ME) and maintained at the Food and Drug Administration animal care facility and treated in accordance and guidelines of the Animal Care and Use Committee. For all experiments, mice were housed with five cage companions in a room set at 72°F and 30*−*70% humidity with a 12 hour light/dark cycle. Infection was introduced in a donor mouse by injection of thawed parasites generated from an uncloned line of *P*. *berghei* ANKA (*Pb−A*) parasites. When parasite burden in the donor mouse reached approximately 5%, blood was collected and diluted in phosphate buffered saline (PBS) to 10^7^ *Pb−A* parasites/ml. Infection was then induced in experimental mice by intraperitoneal injection of 10^6^ *Pb−A* parasites (100 μl). To alleviate suffering, all mice were monitored for clinical systems of ECM twice per day as previously described [27, 28] [29] and euthanized when the first clinical signs of ECM appeared. Mice were euthanized by cervical dislocation under anesthesia (ketamine and xylazine) or carbon dioxide asphyxiation.

### Flow cytometry

Expression of the T cell markers (TCRβ and CD3ε) and coreceptors (CD4 and CD8) on macrophages was measured by flow cytometric analysis of splenocytes and brain sequestered leukocytes (BSLs) using TCRβ-FITC, CD3ε-PE, CD4-PerCP, CD4-PerCP/Cy5.5, CD8-APC/Cy7, CD11b-APC, CD14-PE/Cy7, and F4/80-Brilliant Violet 421™ antibodies purchased from Biolegend (San Diego, CA). Importantly, fluorescence minus one (FMO) controls were used for gating purposes for the TCRβ-FITC, CD3ε-PE, CD11b-APC, CD14-PE/Cy7, and F4/80-Brilliant Violet 421™ antibodies. The mouse Vβ TCR Screening Panel (BD Biosciences, San Jose, CA) was used to determine the type of Vβ TCR expressed by splenic and brain sequestered macrophages. Using this kit, splenocytes and BSLs were stained with CD3ε-PE, CD4-PerCP/Cy5.5, CD8-APC/Cy7, CD11b-APC, CD14-PE/Cy7, and F4/80-Brilliant Violet 421™ antibodies and one of 15 pre-diluted FITC-conjugated monoclonal antibodies specific for mouse Vβ 2, 3, 4, 5.1 and 5.2, 6, 7, 8.1 and 8.2, 9, 10^b^, 11, 12, 13, 14, and 17^a^ T cell receptors. A FITC FMO control was used to determine the appropriate gate for all Vβ TCRs.

Single cell suspensions of splenocytes and brain sequestered leukocytes (BSLs) were prepared as previously described [30, 31] and then stained for 30 minutes with fixable viability dye eFluor^®^506 (eBioscience, San Diego, CA) to distinguish live from dead cells, pre-incubated for 15 minutes with TruStain fcX™ antibody (Biolegend) to block Fc receptors, and labeled for 30 minutes with a panel of appropriately titrated antibodies in Hanks’ Balanced Salt Solution (HBSS) containing 1% BSA. Events were acquired on a standard four laser LSRFortessa X-20™ flow cytometer with FACSDiva 8.0 acquisition software (BD Biosciences, San Jose, CA). Resulting data was analyzed using Flowjo software (Treestar, Ashland, OR).

### AMNIS imaging flow cytometry

BSLs purified from perfused brain tissue of moribund C57BL/6 mice on day 6 post-infection were stained with TCRβ-FITC, CD3ε-PE, CD4-PerCP, CD4-PerCP/Cy5.5, CD8-APC/Cy7, CD11b-APC, CD14-PE/Cy7, and F4/80-Brilliant Violet 421™ antibodies purchased from Biolegend (San Diego, CA) and fixable viability dye eFluor^®^506 (eBioscience, San Diego, CA). An Amnis^®^ ImageStream^®X^ MKII (Amnis Merck-Millipore, Seattle, WA) equipped with five lasers (355, 405, 488, 561, and 642nm) was used for acquisition. A minimum of 30,000 events were collected at 40X magnification using the INSPIRE^®^ software (Amnis Merck-Millipore, Seattle, WA). Analysis was performed using IDEAS® 6.1 software (Amnis Merck-Millipore, Seattle, WA). Focused events were first gated using the Gradient RMS feature. Singlets were then gated by aggregate discrimination using aspect ratio and area. Analysis was then performed on gated live CD11b^high^CD14^+^F480^+^TCRβ^+^CD3ε*−*CD4*−*CD8*−* cells.

### Microscopy

For confocal microscopy, 5 x 10^5^ pooled BSLs purified from perfused brain tissue of moribund C57BL/6 mice on day 6 post-infection were seeded in a 96 well plate, fixed in 4% paraformaldehyde for 30 minutes at room temperature, washed three times with PBS, blocked with 10% goat serum for 30 minutes at room temperature, and centrifuged to remove blocking solution. BSLs were then incubated with 100 μl of primary antibodies (rat anti-mouse F4/80 clone BM8 and Armenian hamster anti-mouse TCRβ clone H57-597) or their respective isotype controls (rat IgG2a, κ, and Armenian hamster IgG) diluted in 2% goat serum to the appropriate concentration for one hour at room temperature. After incubation with primary antibodies, BSLs were washed three times with PBS and then incubated for 45 minutes at room temperature protected from light with secondary antibodies (Alexa Fluor^®^ 594 AffiniPure Goat Anti-Rat IgG [H + L] and Alexa Fluor^®^ 488 AffiniPure Goat Anti-Armenian Hamster IgG [H + L]) diluted 1:300 in 2% goat serum. After incubation with secondary antibodies, BSLs were washed three times, counterstained with 50 μl of Hoecsht 33258 (2 μg/ml) for two minutes, and washed three times with PBS. BSLs were then transferred to ibidi μslides (ibidi, Madison, WI) and mounted with Vectashield (Vector Laboratories, Burlingame, CA). Primary antibodies were purchased from eBioscience (San Diego, CA) or BD Biosciences (San Jose, CA) and secondary antibodies were purchased from Jackson ImmunoResearch Laboratories, Inc. (West Grove, PA). Confocal microscopy was performed using the Leica TCS SP8 DMI6000 microscope system (Leica Microsystems, Manheim, Germany) with a 100x NA1.4 objective lens. Excitation wavelengths of 405, 488 and 594 nm were used for Hoeschst 33248, Alexa Fluor 488 and 594 dyes, respectively. Images were acquired in lif format and stored until analysis. Leica LAS AF and Imaris (Bitplane, South Windsor, CT) software were used for visualization and image analysis.

For light microscopy of TCRβ−expressing macrophages versus TCRβ^+^CD3ε^+^ lymphocytes, cells were sorted from spleen tissue on day 6 post-infection with *Pb*−*A* to ≥ 98% purity on a FACSAria™ Fusion cell sorter (BD Biosciences, San Jose, CA), stained with 5% Giemsa for 15 minutes, and visualized using a DMi8 microscope (Leica Microsystems, Manheim, Germany) with a 100x objective lens.

### PrimeFlow™ RNA assay

Transcript levels of the mouse T cell receptor beta, constant region 1 (TRBC1) gene were measured in naïve and infected (day 6) wildtype, nude and *rag1* KO mice on the C57BL/6 background using the PrimeFlow™ RNA Assay (eBiosciences, Santa Clara, CA). Splenocytes isolated from naïve or *Pb*−*A* infected mice were treated with ACK buffer (Gibco^®^ by Life Technologies) to remove erythrocytes, stained with efluor 506 viability dye (eBiosciences), incubated with TruStain FcX™ (anti-mouse CD16/32) (Biolegend, San Diego, CA), and then stained with antibodies specific for extracellular monocyte/macrophage markers (CD11b, CD14, and F4/80) and T cell markers (TCRβ and CD3ε). To prepare cells for target probe hybridization, cells were fixed with Fixation Buffer 1 at 4°C for 30 minutes, washed 3 times with Permeabilization Buffer containing RNase Inhibitors, fixed again with Fixation Buffer 2 at room temperature for 60 minutes, and then washed 3 times. Cells were then incubated with a target probe specific for the *trbc1* gene at 40°C in a hybridization oven for 2 hours. Signal was then amplified in three sequential hybridization steps: 1) cells were incubated with pre-amplifier DNA (which hybridizes to the target probe) at 40°C for 1.5 hours 2) incubated with amplifier DNA (which hybridizes to the pre-amplifier molecules) at 40°C for 1.5 hours and 3) incubated with label probe oligonucleotide (which hybridizes to the amplifier molecules) conjugated to the Alexa Fluor^®^ 647 fluorescent dye at 40°C for 1 hour. When possible, a minimum of 200,000 lymphocyte events were acquired on an LSRFortessa™ X-20 (BD Biosciences, San Jose, CA).

### Unbiased molecular analysis of TCR expression by macrophages

CD11b^high^CD14^+^F4/80^+^TCRβ^+^CD3ε^−^ macrophages were sorted to ≥98% purity from perfused brain tissue of moribund C57BL/6 mice on day 6 post-infection with *Pb−A* using a FACSAria™ II cell sorter (BD Biosciences, San Jose, CA), snap frozen in RNAlater™, and stored at *−*80°C until use. Characterization of all expressed TCR gene products within brain sequestered macrophages was then performed as previously described [32]. The Oligotex Direct mRNA Mini Kit (Qiagen,Valencia, CA) was used to purify mRNA from TCRβ*−*expressing macrophages. The SMARTer™ PCR cDNA Synthesis Kit (Clontech, Mountain View, CA) was then used to synthesize cDNA from template mRNA. PCR amplification of rearranged TCR products was next performed using the MuMBC primer (TGGCTCAAACAAGGAGACCT) specific for mouse TRBC (Eurofins, Huntsville, AL). TCR gene products were then run on a 1% agarose gel and amplicons that were 500*−*700 bp were extracted from the gel and purified using the NucleoSpin^®^ Extract II kit (Clonetech, Mountain View, CA). Ligation of each TCR gene product into the pGEM-T Easy Vector was then performed followed by transformation into competent *E*. *coli* cells. After transformation, a minimum of 50 colonies were selected for colony PCR using the commercially obtained M13F (TTTTCCCAGTCACGAC) and M13R (CAGGAAACAGCTATGAC) primers (Eurofins, Huntsville, AL). Amplified products that were confirmed appropriate in size by gel electrophoresis were then sequenced using the MuMBC primer. All sequence alignments were performed using the IMGT/V-QUEST alignment tool for TCR nucleotide sequences.

### Phagocytosis assay

An *in vitro* phagocytosis assay was performed to assess the effect of TCRβ on the phagocytic capacity of the macrophage using a 1:1 ratio of splenocytes (labeled with antibodies specific for macrophage and T cell markers) and pRBC (labeled with CellTrace™ Far Red (Invitrogen, Carlsbad, CA)) both isolated from day 3 infected C57BL/6 mice. 10^5^ antibody labeled splenocytes and 10^5^ CellTrace™ labeled pRBC were mixed together and incubated at 37°C for90 minutes. Samples were washed once with PBS and then acquired on a FACSCANTO II flow cytometer (BD Biosciences, San Jose, CA). Phagocytosis of CellTrace™ Far Red pRBC by TCRβ^+^ versus TCRβ*−* Ly6G*−*CD11b^high^F4/80^+^ macrophages was compared using FlowJo software. A fluorescence minus one control was used for gating purposes to distinguish TCRβ^+^ from TCRβ*−* macrophages.

### Microarray expression analysis

To measure the effect of TCRβ expression on the macrophage transcriptome, microarray was performed in quadruplicate on RNA isolated from TCRβ^+^ versus TCRβ*−* CD3ε*−*Ly6G*−*CD11b^high^F4/80^+^CD3ε*−*CD4*−*CD8*−*macrophages purified by fluorescence-activated cell sorting of splenocytes pooled from day 3 *Pb−A* infected C57BL/6 mice using a FACSAria™ Fusion cell sorter (BD Biosciences, San Jose, CA). RNA isolation, amplification, and incorporation of cy3 or cy5 dyes was performed as previously described [33]. Briefly, for preparation of RNA, samples snap frozen in TRIzol^®^ were thawed and RNA was extracted with chloroform, precipitated with isopropanol, washed with 75% ethanol, and resuspended in water. 100 ng of template RNA was then amplified and labeled with cy3 (TCRβ*−*) or cy5 (TCRβ^+^) dyes using the Low Input QuickAmp Labeling Kit (Agilent Technologies, Santa Clara, CA). Labeled cRNA transcripts were then washed twice to remove unincorporated label and resuspended in RNase-free water using the RNeasy® Mini Kit (Qiagen, Valencia, CA) and equivalent amounts were hybridized to a custom designed murine oligonucleotide chip containing 37558 probes 60 base pairs in size (Agilent Technologies, Santa Clara, CA). This microarray dataset has been deposited into the Gene Expression Omnibus public functional genomics data repository (GEO accession number GSE111593). Data was filtered as previously described [34] using NIAID microarray database tools (mAdb.niaid.nih.gov) with a criteria of 1) fold change ratio of ± 3 in four arrays and 2) p value < 0.01.

### Statistical analysis

Two-way ANOVA followed by Bonferroni was used to measure significant differences in the proportion, absolute number, and MFI of TCRαβ on the macrophage between C57BL/6 versus Balb/c mice over the course of a *Pb−A* infection. The Mann Whitney *U* test was applied to detect differences in the percent expression and MFI of TCRαβ on CD11b^high^, CD11b^high^CD14^+^, CD11b^high^F4/80^+^, and CD11b^high^CD14^+^F4/80^+^ cells. The Student’s *t* test was used to compare the size of TCRβ^+^CD11b^high^CD14^+^F4/80^+^ cells to TCRβ^+^CD3ε^+^CD4^+^ and TCRβ^+^CD3ε^+^CD8^+^ cells by Amnis^®^. The Student’s *t* test was also used to compared the diameter (μm) of Giemsa stained TCRβ^+^CD3ε*−*CD11b^high^CD14^+^F4/80^+^ cells versus TCRβ^+^CD3ε^+^ lymphocytes sorted from spleen tissue of C57BL/6 mice on day 6 post-infection with *Pb−A*.

## Results

### There are two distinct subsets of TCRβ^+^ brain-sequestered leukocytes during ECM

**Fig 1:**
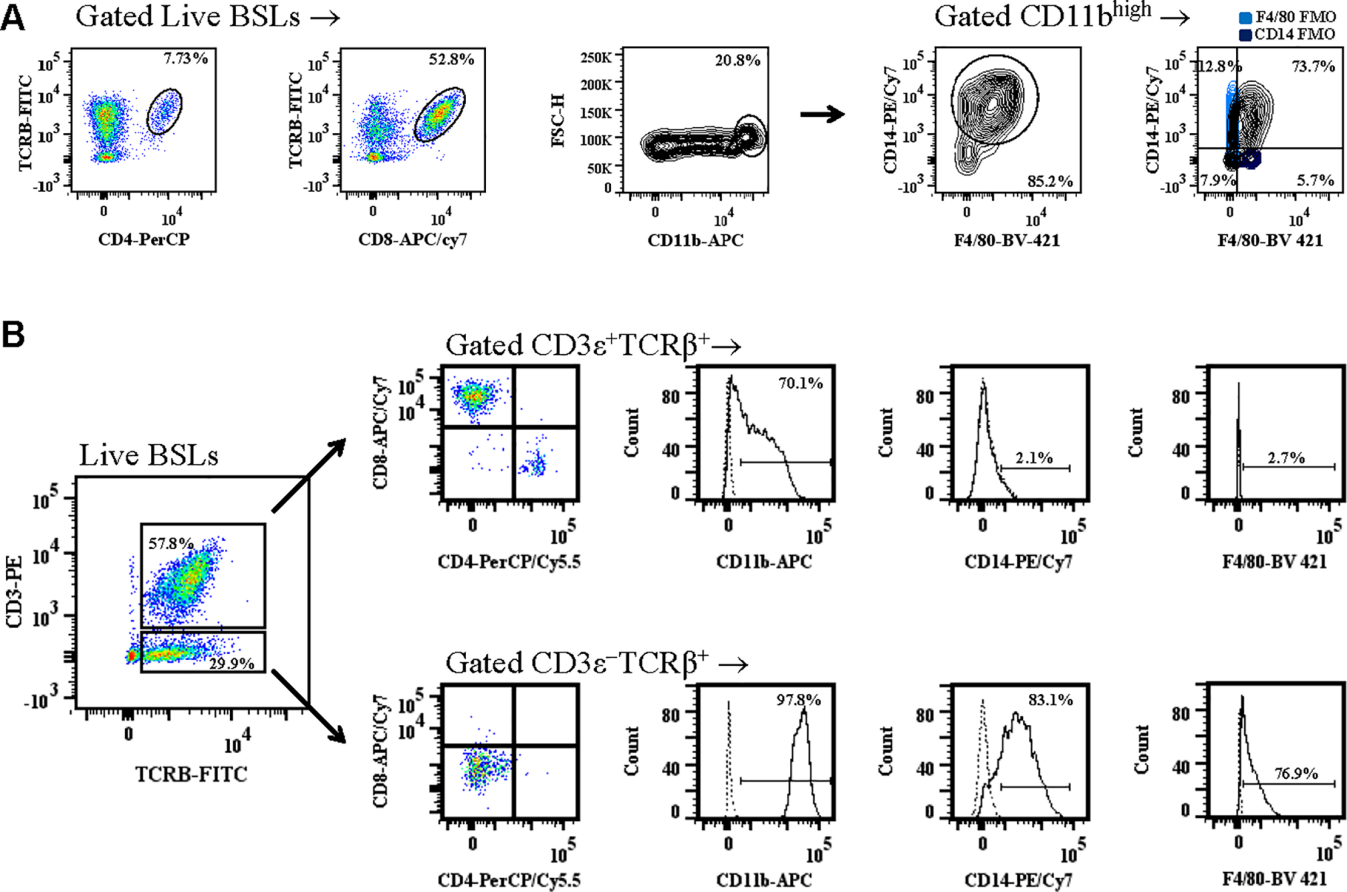
Subsets of brain sequestered leukocytes during experimental cerebral malaria. C57BL/6 mice were infected with 10^6^ *Plasmodium berghei* ANKA parasites and brain sequestered leukocytes **(**BSLs) were purified from perfused brain tissue of moribund mice (n = 5). *A*: CD4^+^ T cells, CD8^+^ T cells and macrophages were then quantitated by flow cytometric analysis. FMO controls for CD14 (purple) and F4/80 (light blue) were used to determine the proportion of CD11b^high^ brain sequestered leukocytes that express CD14 and F4/80. *B*: Phenotypic analysis of stained BSLs was performed to compare expression of the CD4^+^ and CD8^+^ T cell coreceptors and the CD11b, CD14, and F4/80 monocyte/macrophage lineage markers on TCRβ^+^CD3ε^+^ versus TCRβ^+^CD3ε^-^ BSLs. Fluorescence minus one controls (dashed line) were used for gating purposes for CD11b, CD14, and F4/80 antibody staining. Data presented is representative of three independent experiments.

ECM is characterized by the infiltration of pathogenic immune cells to the brain during the cerebral phase of disease [35]. CD4^+^ T cells, CD8^+^ T cells, and macrophages are the three major subsets of immune cells that infiltrate the brain during ECM. We found that CD4^+^ T cells were 8.6 ± 1.3%, CD8^+^ T cells were 54.7 ± 6.5%, and CD11b^high^ macrophages coexpressing CD14 and/or F4/80 were 10.3 ± 2.5% (Fig 1*A*) of brain-sequestered leukocytes (BSLs) purified from perfused tissue of moribund mice. Experiments using fluorescence minus one (FMO) controls for CD14 and F4/80 demonstrated that the majority (73.7%) of brain sequestered CD11b^high^ cells were triple positive CD11b^high^CD14^+^F4/80^+^ macrophages (Fig 1*A*).

Interestingly, when coexpression of the T cell markers CD3ε and TCRβ was measured on brain sequestered leukocytes (BSLs) of mice with ECM, there were two distinct populations of TCRβ^+^ cells distinguished by the presence or absence of CD3ε; 57.8% of live BSLs were TCRβ^+^CD3ε^+^ and 29.9% percent of live BSLs were TCRβ^+^CD3ε*−* (Fig 1*B*). Brain sequestered TCRβ^+^CD3ε^+^ cells coexpressed either the CD4 or CD8 receptor. In contrast, all TCRβ^+^CD3ε*−* cells were CD4*−*CD8*−*. In an experiment using FMO controls for gating purposes, we examined monocyte/macrophage lineage markers on TCRβ^+^CD3ε^+^ versus TCRβ^+^CD3ε*−* BSLs. Although the majority of TCRβ^+^CD3ε^+^ cells express CD11b, both a cell surface integrin of macrophages and a marker of CD8^+^ T cell memory [36], this cell population did not express the CD14 or F4/80 monocyte/macrophage markers. In contrast, brain sequestered TCRβ^+^CD3ε^−^ cells expressed CD11b, CD14, and F4/80. These results suggest that the TCRβ^+^CD3ε^+^ cells are classical T cells whereas the TCRβ^+^CD3ε^−^ cells belong to the monocyte/macrophage lineage.

### Brain sequestered macrophages express high levels of TCRβ during ECM

**Fig 2:**
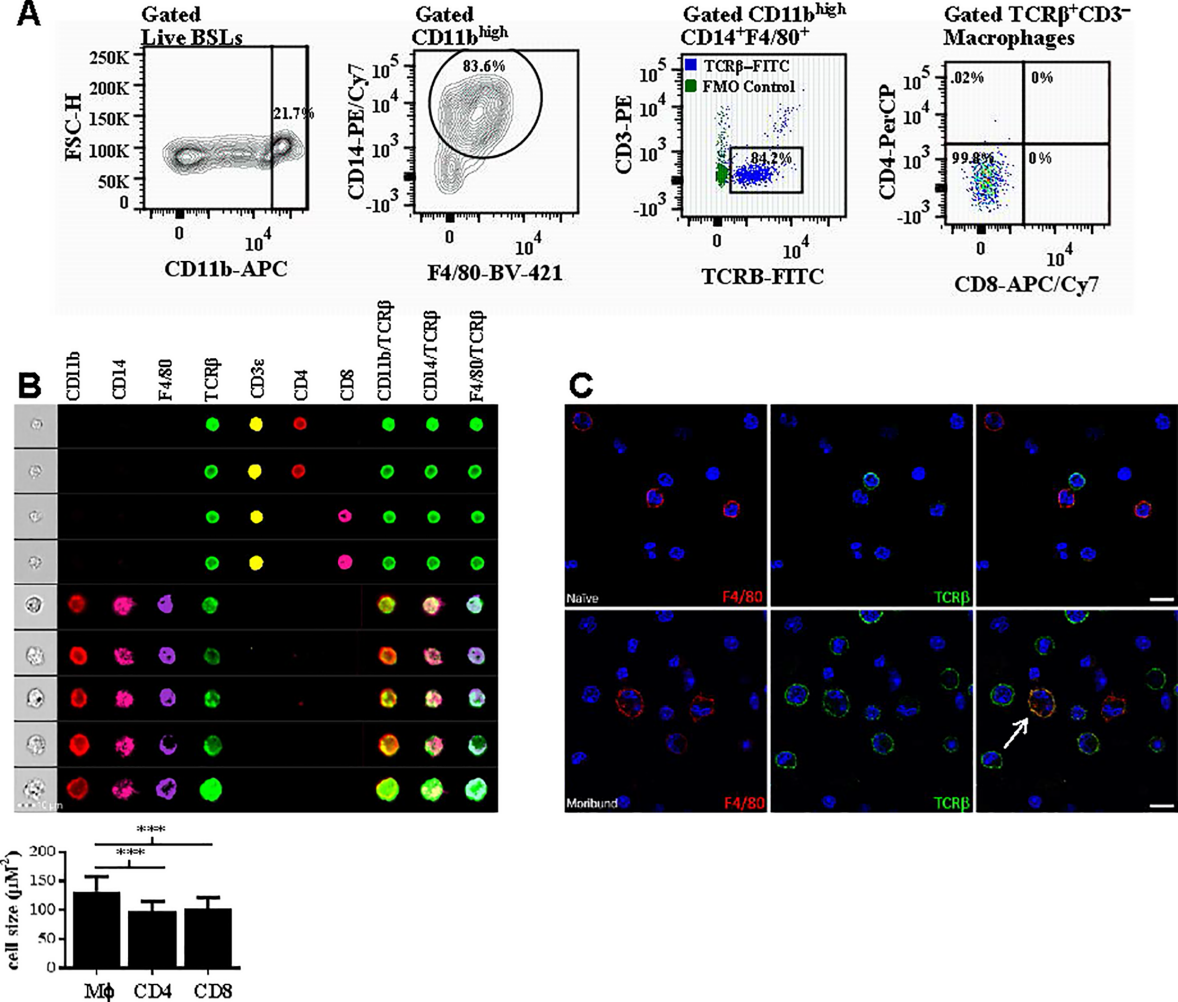
Brain sequestered macrophages express high levels of TCRβ. *A*: Brain sequestered leukocytes (BSLs) purified from perfused brain tissue of moribund mice (n = 4) with experimental cerebral malaria on day 6 post-infection with *Plasmodium berghei* ANKA were stained for flow cytometric analysis. An FMO control (green) was used for gating purposes to determine the proportion of brain sequestered macrophages that express TCRβ (blue). *B*: CD11b^high^CD14^+^F4/80^+^ macrophages that were TCRαβ^+^CD3ε*−* and CD4*−*CD8*−* were gated from live singlet BSLs purified from perfused brain tissue of moribund mice on day 6 post-infection and colocalization of TCRβ with the CD11b, CD14, and F4/80 monocyte/macrophage lineage markers was visualized on individual cells by AMNIS imaging. In addition, measurement of cell size by AMNIS imaging demonstrates that TCRβ expressing CD11b^high^CD14^+^F4/80^+^ cells (n = 314) are significantly larger than CD4^+^TCRβ^+^CD3ε^+^ (n = 102) and CD8^+^TCRβ^+^CD3ε^+^ (n = 586) lymphocytes. *C*: Brain sequestered leukocytes (BSLs) purified from perfused brain tissue of naïve or moribund C57BL/6 mice on day 6 post-*Pb−A* infection were stained with the pan-macrophage marker F4/80 (red) and TCRβ (green) and counterstained with DAPI (blue). Cellular colocalization of TCRβ with F4/80 was observed by confocal microscopy on BSLs from moribund but not naïve mice. Importantly, isotype controls for F4/80 (rat IgG2a, κ) and TCRβ (Armenian hamster IgG) stained negative in both moribund and naïve mice. Significant differences between macrophage versus CD4^+^ and CD8^+^ T lymphocyte cell size are indicated as ****P < 0.001. P*−*values were calculated using the Student *t* test. Error bars indicate standard deviation.

Next, we quantitated the expression of TCRβ on brain sequestered macrophages purified from moribund mice with ECM (n = 4). 16.9 ± 2.3% of live BSLs were CD11b^high^ (Fig 2*A*) and the majority (70.4 ± 8.0%) of these CD11b^high^ BSLs were macrophages coexpressing CD14 and/or F4/80. Remarkably, using this gating strategy to define macrophages and an FMO to set the TCRβ gate, we found that 84.2 ± 2.3% (5.48 ± 1.83 x 10^4^) of brain sequestered macrophages were TCRβ^+^CD3ε*−*. The MFI of TCRβ-FITC on these macrophages was 1856 ± 180. Lastly, 99.8% of TCRβ*−*expressing macrophages were CD4*−*CD8*−*. S1 Fig depicts the complete gating strategy used to identify this novel population of cells.

We applied Amnis^®^ imaging flow cytometry to visualize colocalization of TCRβ with the CD11b, CD14, and F4/80 monocyte/macrophage lineage markers on gated live CD11b^high^CD14^+^F4/80^+^ macrophage singlets that were TCRβ^+^CD3ε*−* and CD4*−*CD8*−* (Fig 2*B*). Using this approach, we found that TCRβ was coexpressed with CD11b, CD14, and F4/80 on single cells. We also used Amnis^®^ imaging to compare the size of TCRβ^+^CD11b^high^CD14^+^F4/80^+^ cells to CD4^+^ and CD8^+^ T lymphocytes; these cells (131 ± 27 μM^2^) were significantly larger (p<0.0001, Student’s t test) than TCRβ^+^CD3ε^+^CD4^+^ (99 ± 16 μM^2^) and TCRβ^+^CD3ε^+^CD8^+^ (103 ± 19 μM^2^) lymphocytes suggesting that TCRβ^+^CD11b^high^CD14^+^F4/80^+^ cells are macrophages expressing TCRβ rather than T lymphocytes expressing monocyte/macrophage markers. Lastly, we used IDEAS^®^ software (MilliporeSigma, Burlington, MA) to quantitate the level of TCRβ on a given cell and found that the intensity of TCRβ expression on CD11b^high^CD14^+^F4/80^+^ macrophages (49,312 ± 10015 pixels) was similar to the intensity of TCRβ expression on CD3ε^+^CD4^+^ (44033 ± 4067 pixels) and CD3ε^+^CD8^+^ (34641 ± 1592 pixels) T lymphocytes. In addition to utilizing Amnis^®^ imaging to observe colocalization, we performed confocal microscopy on BSLs purified from perfused tissue of mice with ECM (Fig 2*C*). BSLs from naïve versus moribund mice were stained with antibodies specific for the pan−macrophage marker F4/80 and TCRβ and colocalization was then measured on merged images. Importantly, double positive F4/80^+^TCRβ^+^ cells were observed in the BSL population isolated from moribund but not naïve mice. However, isotype controls for F4/80 (rat IgG2a, κ) and TCRβ (Armenian hamster IgG) stained negative in both moribund and naïve mice. In summary, our results from flow cytometry of monocyte/macrophage and T cell markers and imaging with Amnis^®^ technology and by confocal microscopy indicate that a novel population of TCRβ-expressing CD11b^high^CD14^+^F4/80^+^ macrophages sequester in the brain during ECM.

### Pb−A malaria stimulates rapid expansion of TCRβ-expressing CD11b^high^CD14^+^F4/80^+^ macrophages in the spleen

**Fig 3:**
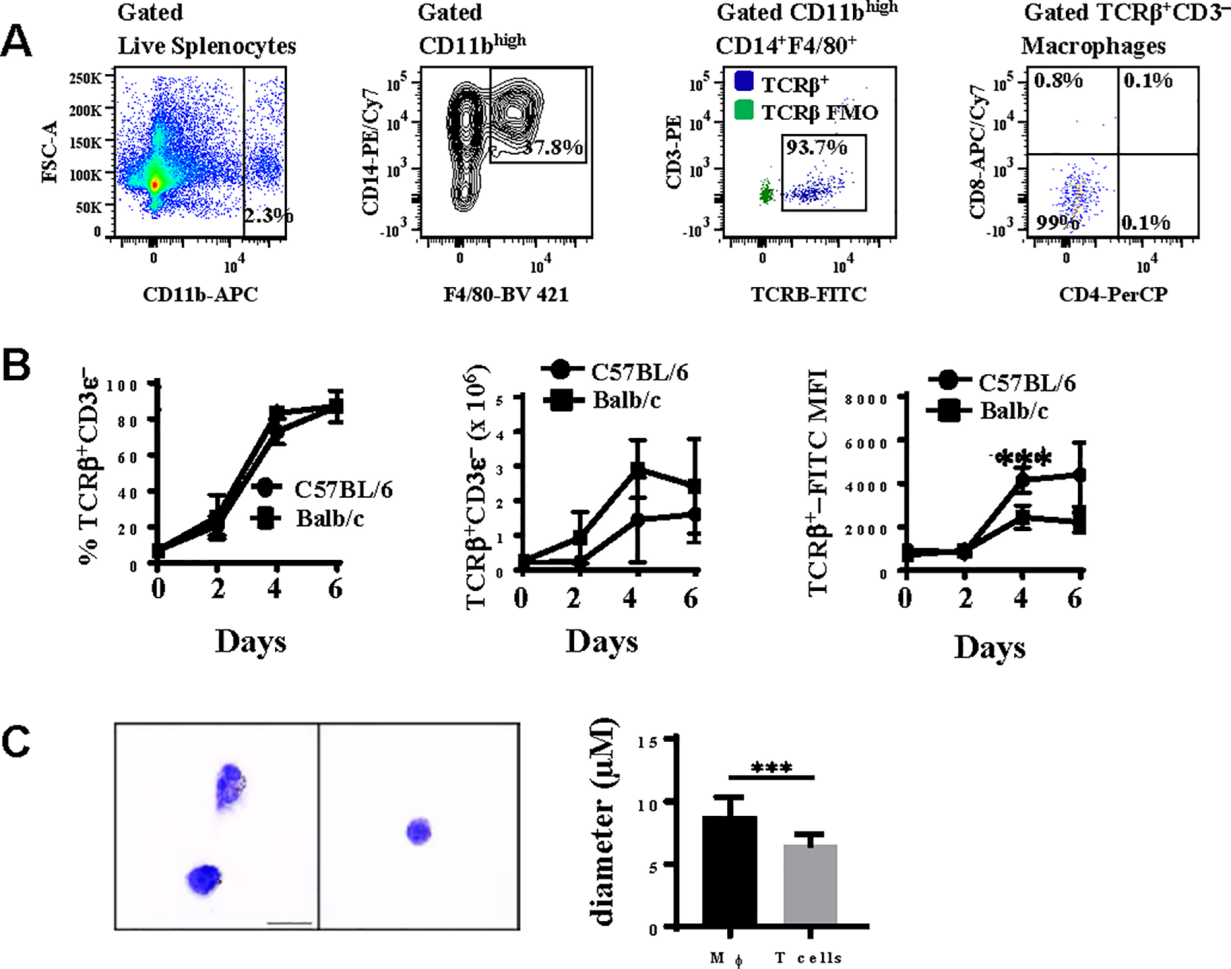
TCRβ expression on splenic macrophages during *Plasmodium berghei* ANKA malaria. *A*: Splenic CD11b^high^CD14^+^F4/80^+^ macrophages were quantitated by measuring the coexpression of CD14 and F4/80 on CD11b^high^ cells gated from live lymphocytes isolated from spleen tissue. Using a TCRβ-FITC fluorescence minus one (FMO) control for gating purposes, the proportion of CD11b^high^CD14^+^F4/80^+^ macrophages that were TCRβ^+^CD3ε**−** was then determined. TCRβ-expressing macrophages were also analysed for the expression of CD4 and CD8. *B*: The percentage, absolute number, and mean fluorescence intensity (MFI) of TCRβ on splenic CD11b^high^CD14^+^F4/80^+^ macrophages was enumerated and compared in naïve and *Pb***−***A* infected C57BL/6 and Balb/c mice on days 2, 4, and 6 post-infection (n = 6 per time point for each strain of mouse). *C*: TCRβ^+^CD3ε**−**CD11b^high^CD14^+^F4/80^+^ cells and TCRβ^+^CD3ε^+^ lymphocytes were sorted from spleen tissue on day 6 post-infection with *Pb***−***A* and stained with Giemsa to compare the size and morphology of each cell type. Cell size was compared by measuring the diameter (μm) of TCRβ^+^CD3ε**−**CD11b^high^CD14^+^F4/80^+^ cells (n = 60) versus TCRβ^+^CD3ε^+^ lymphocytes (n = 60). Data shown is representative of three independent experiments. Significant differences between C57BL/6 and Balb/c mice are indicated as ***P < 0.001. P**−**values were calculated using two-way ANOVA followed by Bonferroni. Error bars indicate standard deviation. Significant difference in cell size (diameter) between TCRβ^+^CD3ε**−**CD11b^high^CD14^+^F4/80^+^ cells and TCRβ^+^CD3ε^+^ lymphocytes is indicated as ***P < 0.001. P−value was calculated using Student’s *t* test. Error bars indicate standard deviation.

Since the majority of brain sequestered macrophages are CD11b^high^CD14^+^F4/80^+^ during ECM (Fig 1*A*), we next examined the expression of TCRβ on CD11b^high^CD14^+^F4/80^+^ macrophages in spleen tissue of ECM-susceptible C57BL/6 versus ECM-resistant Balb/c mice over the course of a *Pb***−***A* infection. The complete gating strategy used to identify TCRβ expression on CD11b^high^CD14^+^F4/80^+^ macrophages in the spleen is shown in S2 Fig. CD11b^high^ cells were first gated from live lymphocytes (Fig 3*A*) and these CD11b^high^ cells were then selected for CD14 and F4/80 coexpression. TCRβ and CD4 and CD8 expression were then measured on the CD11b^high^CD14^+^F4/80^+^ macrophages. A small population (2.2 ± 0.6 x 10^5^) of CD11b^high^CD14^+^F4/80^+^ macrophages expressed TCRβ in naïve mice (Fig 3*B*). However, TCRβ**−**expressing CD11b^high^CD14^+^F4/80^+^ macrophages increased significantly over the course of a *Pb***−***A* infection; in ECM susceptible C57BL/6 mice, the number of TCRβ**−**expressing CD11b^high^CD14^+^F4/80^+^ macrophages increased 6.5 fold (p<0.01, Mann Whitney *U* test) from day 2 (2.2 x 10^5^) to day 4 (1.4 ± 0.5 x 10^6^) post-infection and the percentage of CD11b^high^CD14^+^F4/80^+^ macrophages that express TCRβ was maximal (87.0 ± 3.6%) on day 6 post-infection when mice were exhibiting symptoms of ECM. Although there was no significant difference between the percentage or absolute number of CD11b^high^CD14^+^F4/80^+^ macrophages that express TCRβ between ECM-susceptible C57BL/6 mice and ECM-resistant Balb/c mice over the course of a *Pb***−***A* infection, the mean fluorescence intensity (MFI), a measure of the protein quantity per cell, of TCRβ-FITC was significantly higher (1.7 fold, p<0.0001, Two-way ANOVA followed by Bonferroni) in C57BL/6 (4159 ± 241) compared to Balb/c (2453 ± 224) mice on day 4 post-infection.

We also sorted TCRβ^+^CD3ε**−**CD11b^high^CD14^+^F4/80^+^ cells and TCR^+^CD3ε^+^ lymphocytes from spleen tissue on day 6 post-infection with *Pb−A* and compared the size and morphology of these cells by microscopy (Fig 3*C*). TCRβ^+^CD3ε^−^CD11b^high^CD14^+^F4/80^+^ cells (8.86 ± 0.19 μm) were significantly larger (p<0.001, Student’s *t* test) in diameter than TCR^+^CD3ε^+^ lymphocytes (6.49 ± .11 μm). In addition, numerous TCRβ^+^CD3ε^−^CD11b^high^CD14^+^F4/80^+^cells had phagocytosed *Pb−A* parasite(s). Together, these results indicate that TCRβ^+^CD3ε^−^CD11b^high^CD14^+^F4/80^+^ cells are macrophages expressing TCRβ rather than T lymphocytes expressing macrophage markers.

### Optimal expression of TCRβ on CD11b^high^ immune cells is associated with coexpression of CD14 and F4/80

**Fig 4:**
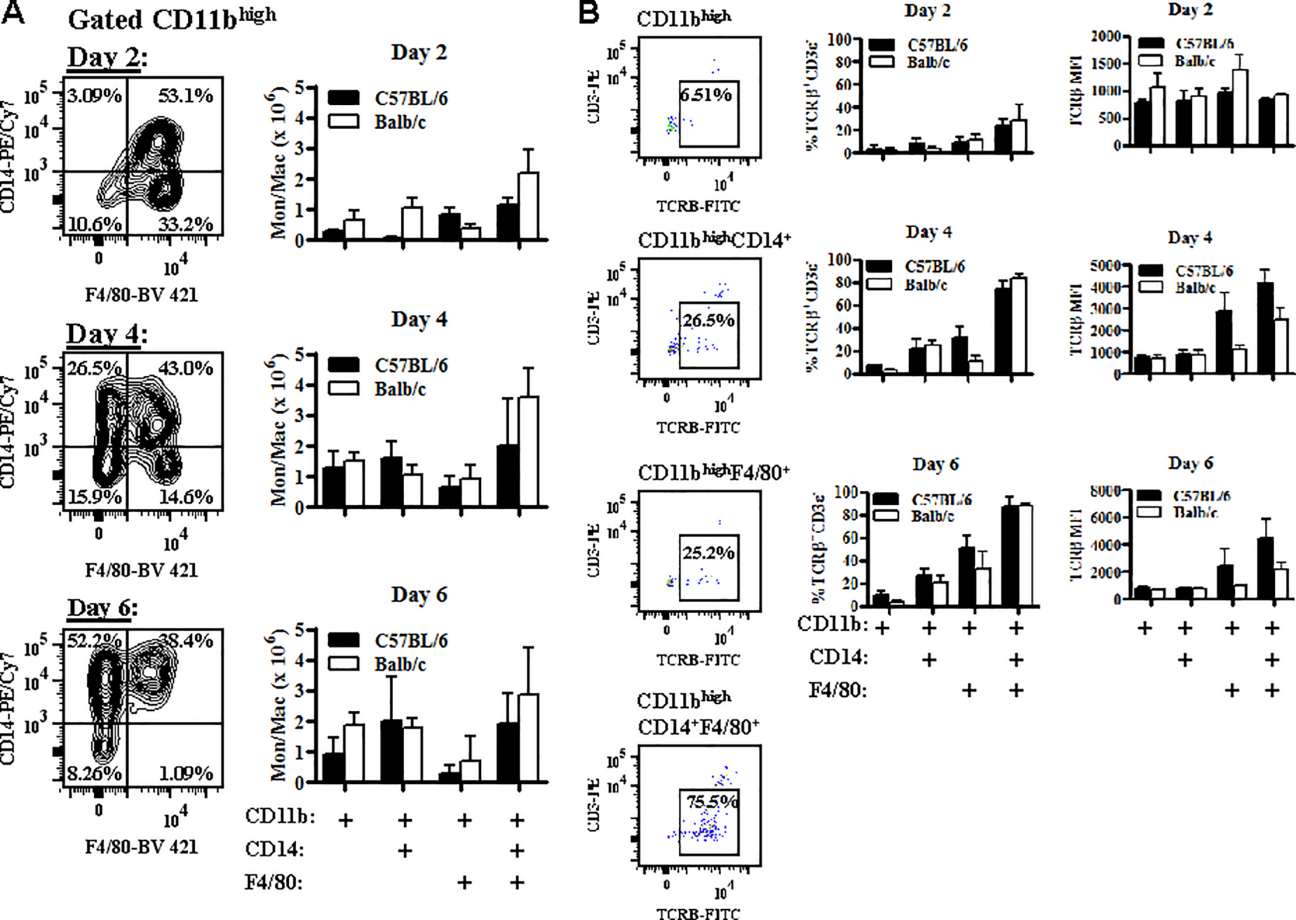
Optimal expression of TCRβ is associated with coexpression of both CD14 and F4/80 on CD11b^high^ cells. *A*: C57BL/6 (n = 6) and Balb/c (n = 6) mice were infected with 10^6^ *Plasmodium berghei* ANKA parasites. CD14 and F4/80 expression was then measured on gated CD11b^high^ splenocytes on days 2, 4, and 6 post-infection and in naïve mice (data not shown) and the absolute number of CD11b^high^, CD11b^high^CD14^+^, CD11b^high^F4/80^+^, and CD11b^high^CD14^+^F4/80^+^ cells was enumerated at each time point. *B*: The effect of CD14 and F4/80 on TCRβ expression on CD11b^high^ splenocytes was also determined in C57BL/6 and Balb/c mice by comparing the expression of TCRβ on CD11b^high^, CD11b^high^CD14^+^, CD11b^high^F4/80^+^, and CD11b^high^CD14^+^F4/80^+^ cells. The percentage of cellular subsets that were TCRβ^+^CD3ε^−^ and the TCRβ-FITC mean fluorescence intensity (MFI) were calculated on days 2, 4, and 6 post-*Pb−A* infection. Data presented is representative of three independent experiments. The absolute number of cellular subsets that were TCRβ^+^CD3ε^−^ can be found in S3 Fig.

In depth analysis of gated CD11b^high^ splenic immune cells suggests that this population is dynamic and changes rapidly over the course of a *Pb−A* infection in susceptible C56BL/6 mice. On day 2 post-infection, the majority of CD11b^high^ cells were CD11b^high^CD14^+^F4/80^+^ (49.1 ± 2.2%) or CD11b^high^F4/80^+^ (35.4 ± 3.0%) macrophages (Fig 4*A*). However, between day 2 and day 4 post-infection, there was a non-significant loss of CD11b^high^F4/80^+^ cells that was accompanied by a significant gain (18.3 fold) in CD11b^high^CD14^+^ cells (p<0.01, Mann Whitney *U* test). From day 4 to day 6 post-infection, CD11b^high^F4/80^+^ cells were further depleted and on day 6 post-infection, the majority of CD11b^high^ cells were CD11b^high^CD14^+^F4/80^+^ (37.1 ± 1.9%) or CD11b^high^CD14^+^ (38.8 ± 5.1%). Hence, while CD11b^high^CD14^+^F4/80^+^ cells remain a major population of gated CD11b^high^ immune cells throughout the course of infection, there is a shift from CD11b^high^F4/80^+^ cells early in infection to CD11b^high^CD14^+^ cells late in infection. Interestingly, this shift was not as prominent in resistant Balb/c mice; although there was a moderate (1.7 fold) but significant (p<0.01, Mann Whitney *U* test) increase in the number of CD11b^high^CD14^+^ cells from day 4 to day 6 post-infection, loss of splenic CD11b^high^F4/80^+^ cells in Balb/c mice over the course of infection was not observed.

Since CD11b^high^F4/80^+^ cells are a major subset early in infection and CD11b^high^CD14^+^ cells are a major subset late in infection in susceptible C57BL/6 mice, we measured the effect of CD14 and F4/80 on TCRβ expression on gated CD11b^high^ cells by comparing the percent expression and MFI of TCRβ on CD11b^high^, CD11b^high^CD14^+^, CD11b^high^F4/80^+^, and CD11b^high^CD14^+^F4/80^+^ cells on days 2, 4, and 6 post-*Pb−A* infection (Fig 4*B*). Interestingly, optimal expression of TCRβ is associated with expression of both CD14 and F4/80 on CD11b^high^ cells with maximal differences observed on day 4 post-infection; on day 4 post-infection, the percentage of CD11b^high^CD14^+^F4/80^+^ that were TCRβ^+^CD3ε^−^ was 11.1 fold higher (p<0.0001, Mann Whitney *U* test) than CD11b^high^cells, 3.3 fold higher (p<0.0001, Mann Whitney *U* test) than CD11b^high^CD14^+^ cells, and 2.3 fold higher (p<0.0001, Mann Whitney *U* test) than CD11b^high^F4/80^+^ cells. Similarly, the MFI of TCRβ−FITC on CD11b^high^CD14^+^F4/80^+^ was 5.3 fold higher (p<0.01, Mann Whitney *U* test) than CD11b^high^cells, 4.7 fold higher (p<0.01, Mann Whitney *U* test) than CD11b^high^CD14^+^ cells, and 1.5 fold higher (p<0.05, Mann Whitney *U* test) than CD11b^high^F4/80^+^ cells. Similar results were found in ECM-resistant Balb/c mice.

### Measurement of TCRβ on macrophages isolated from nude and rag1 knockout mice

**Fig 5:**
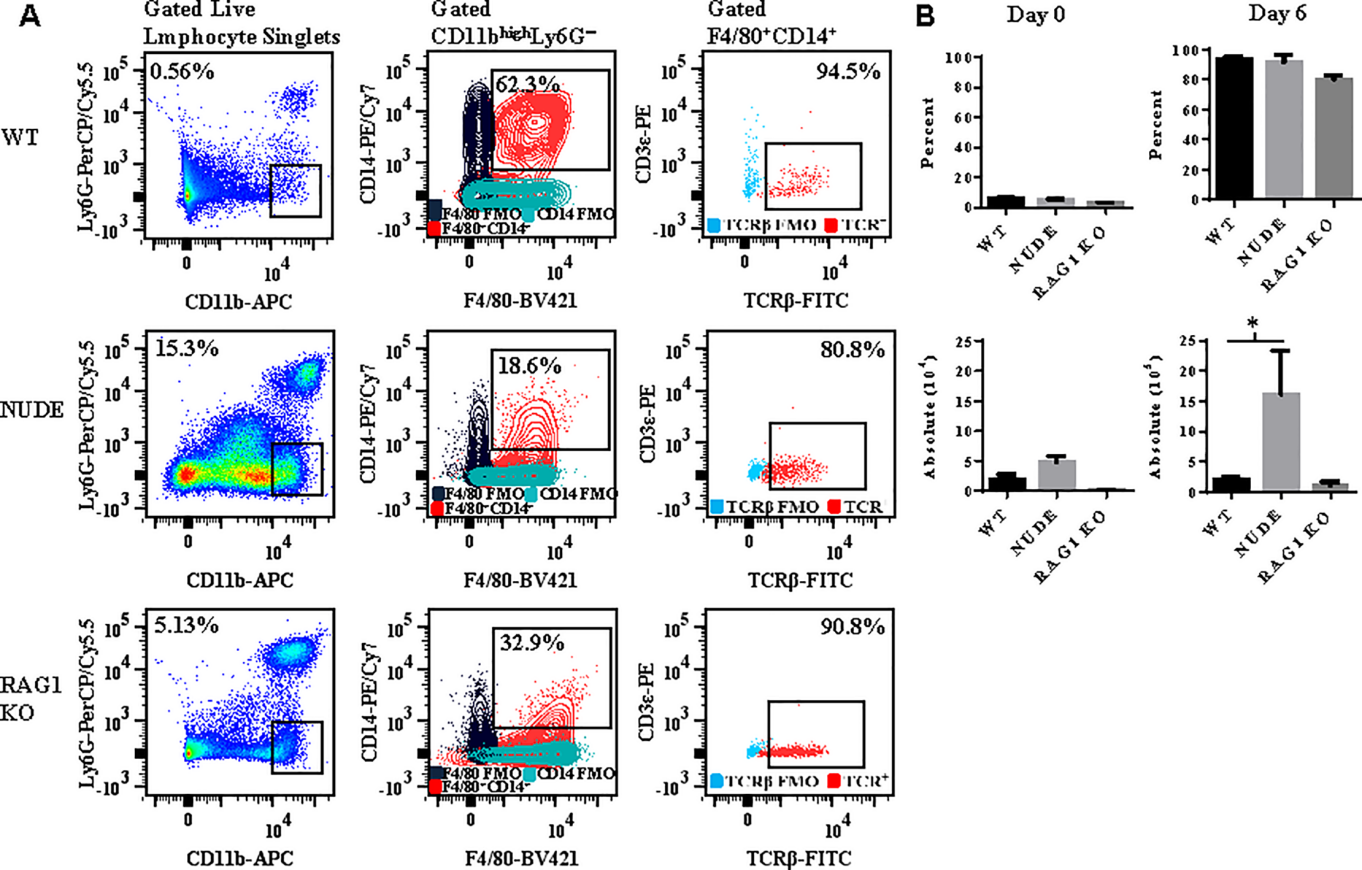
Comparison of TCRβ expression on macrophages in C57BL/6 wildtype verses nude and *rag1* knockout mice. *A*: Wildtype (WT), nude, and *rag1* knockout mice were infected with 10^6^ *Plasmodium berghei* ANKA (*Pb−A*) parasites and TCRβ expression was measured on gated Ly6G^−^CD11b^high^CD14^+^F4/80^+^ macrophages from spleen tissue of the three types of mice. *B*: The percentage of macrophages that were TCRβ^+^CD3ε^−^ and the absolute number of TCRβ^+^CD3ε^−^ macrophages was compared in naïve and *Pb−A* infected (day 6) WT, nude, and *rag1* knockout mice. Results are presented as mean ± standard deviation and are representative of two independent experiments. Significant difference between WT versus nude mice is indicated as *P < 0.05. P−value was calculated using the Mann Whitney *U* test.

To establish that observed TCRβ expression by the macrophage was not a consequence of phagocytosis or trogocytosis (membrane swapping) of peripheral T cells, we compared expression of TCRβ on the surface of Ly6G^−^CD11b^high^CD14^+^F4/80^+^ splenic macrophages in wildtype (WT) versus nude (which lack T cells) and *rag1* KO (which lack T and B cells) mice on the C57BL/6 background (Fig 5*A*). In naïve WT, nude, and *rag1* knockout mice, macrophages expressed low levels of TCRβ (Fig 5*B*). However, by day 6 post-infection with *Pb−A*, the majority (90.8 ± 2.3%) of macrophages expressed TCRβ in moribund WT mice. A similar proportion of macrophages expressed TCRβ in nude and *rag1* knockout mice; 92.3 ± 1.6% of macrophages expressed TCRβ in nude mice and 88.5 ± 0.7% of macrophages expressed TCRβ in *rag1* knockout mice. However, nude mice had significantly more (p<0.05, Mann Whitney *U* test) TCRβ−expressing macrophages compared to WT and *rag1* KO mice due to an abundance of splenic macrophages likely caused by immune compensatory mechanisms. These results demonstrate that the proportion of macrophages that express TCRβ is not increased by the presence of peripheral T cells in WT mice. We note that although expression of high levels of TCRβ protein on macrophages deficient for the *rag1* gene was very unexpected since *rag1* is essential for V(D)J recombination, these results are corroborated by a study demonstrating that neutrophils isolated from recombination-defective SCID mice and X-linked SCID humans express TCRαβ [21].

### Macrophages express abundant levels of TCRβ transcript

**Fig 6:**
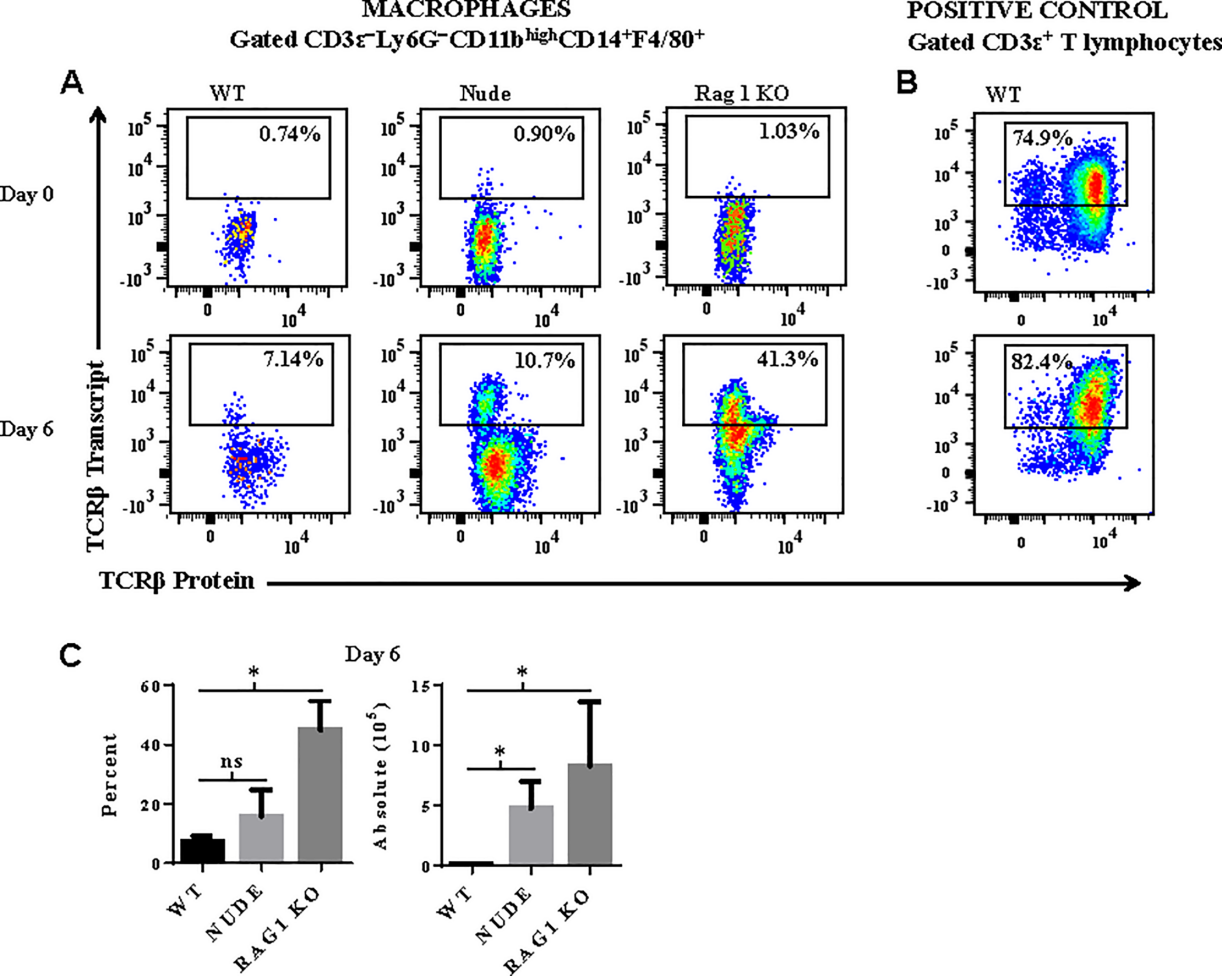
Macrophages express abundant levels of TCRβ transcripts. *A*: Intracellular transcript levels of TCRβ was measured in splenic macrophages isolated from naïve and *Plasmodium berghei* ANKA infected wildtype (WT), nude, and *rag1* knockout (KO) using the flow cytometry based PrimeFlow^®^ RNA assay. *B*: Measurement of TCRβ transcript in positive control CD3ε^+^ T lymphocytes was also performed to verify that the oligonucleotide probe set was specific for TCRβ. *C*: The percentage of macrophages that express TCRβ transcript and the absolute number of TCRβ transcript expressing macrophages are presented as mean ± standard deviation and are representative of two independent experiments. Significant differences between WT versus nude and *rag1* KO mice are indicated as *P < 0.05. P−values were calculated using the Mann Whitney *U* test.

To verify that TCRβ expression by the macrophage was not caused by anti-TCRβ binding to the Fc receptor or a cross-reactive epitope on the macrophage, we measured intracellular transcript levels of mouse T cell receptor beta, constant region 1 (TRBC1) by the flow cytometry based PrimeFlow^®^ RNA assay. This assay has recently been developed to detect RNA using a set of 40 short oligonucleotide probes specific for a target gene. Due to the small size of each oligonucleotide probe, a single nucleotide difference in TRBC1 transcript will prevent hybridization of the probe to TRBC1 RNA. Positive control CD3ε^+^ T lymphocytes isolated from spleen tissue of naïve and *Pb−A* infected WT mice expressed abundant levels of TCRβ transcript (Fig 6*B*). In WT mice, the proportion of macrophages that expressed TCRβ transcript increased from 0.6 ± 0.1% to 7.4 ± 1.0% from day 0 to day 6 post-infection (Fig 6*A* and 6*C*). Similar results were observed in nude mice; the percent of macrophages that express TCRβ transcript increased from 1.2 ± 0.3% (day 0) to 15.7 ± 3.1% (day 6) in nude mice. Remarkably, on day 6 post-infection, 44.9 ± 4.4% of macrophages expressed TCRβ in *rag1* knockout mice. These results demonstrate that *Pb−A* infection induces abundant transcription of TCRβ in macrophages. However, while it is clear that a discrete population of macrophages express TCRβ transcript, we note that the correlation between TCRβ transcript and protein is lower in macrophages compared to positive control CD3ε^+^ T cells. This weaker correlation may be caused by a difference in transcriptional/translational regulation of the TCRβ gene in macrophages versus T cells. Furthermore, the lower levels of TCRβ transcript versus protein that we observed in macrophages is supported by studies demonstrating that mRNAs 1) are produced at a much lower rate and 2) are less stable than their corresponding proteins in mammalian cells [37].

### Macrophage TCRβ undergoes productive gene rearrangements

**Fig 7:**
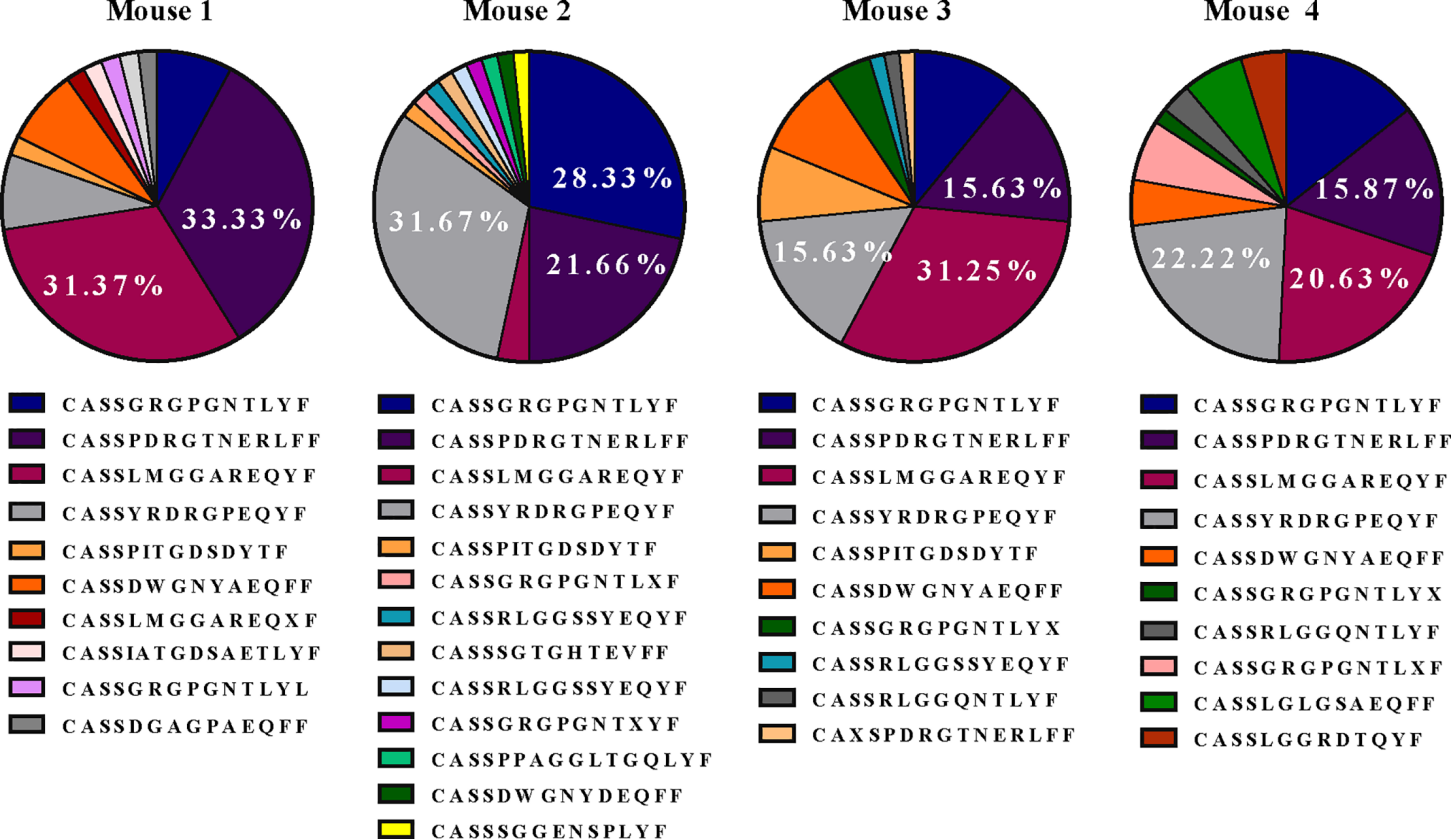
Molecular analysis of T cell receptor expression in brain sequestered macrophages. C57BL/6 mice were infected with 10^6^ *Plasmodium berghei* ANKA parasites and approximately 1500 brain sequestered macrophages of the TCRβ^+^CD3ε^−^CD11b^high^CD14^+^F4/80^+^ phenotype were sorted from perfused brain tissue of four moribund mice with experimental cerebral malaria. Unbiased quantification and characterization of all expressed T cell receptor (TCR) gene products was then performed by sequencing the complementarity-determining region 3 (CDR3) of T cell receptor β transcripts within the macrophage population of each mouse. A minimum of 50 productive gene rearrangements were sequenced per sample. Venn diagrams illustrate a diverse repertoire of expressed TCRβ genes and substantial overlap in preferred usage of TCRβ among the four mice.

To determine if macrophage TCRβ undergoes productive gene rearrangement, we sequenced the complementarity-determining region 3 (CDR3) of T cell receptor β transcripts purified from 1500 sorted brain sequestered macrophages of the TCRβ^+^CD3ε^−^CD11b^high^CD14^+^F4/80^+^ phenotype isolated from four mice with ECM using a published protocol [32]. This molecular method quantifies all expressed T cell receptor transcripts and represents the diversity of the TCRβ repertoire in the population under investigation. Using this approach, we report that the brain sequestered macrophage population in each mouse expressed a repertoire of TCRβ genes that was diverse yet contained a dominant TCRβ CDR3 region indicating preferred usage. Furthermore, we report substantial overlap in this preferred usage of TCRβ transcripts among the four mice (Fig 7). For example, CASSLMGGAREQYF was a dominant productive rearranged sequence in three of four mice. In summary, these results demonstrate that the TCRβ gene undergoes productive rearrangements and expresses a diverse repertoire of transcripts in macrophages during *Pb−A* malaria.

### Brain sequestered CD11b^high^CD14^+^F4/80^+^ macrophages preferentially express the Vβ7 T cell receptor

**Fig 8:**
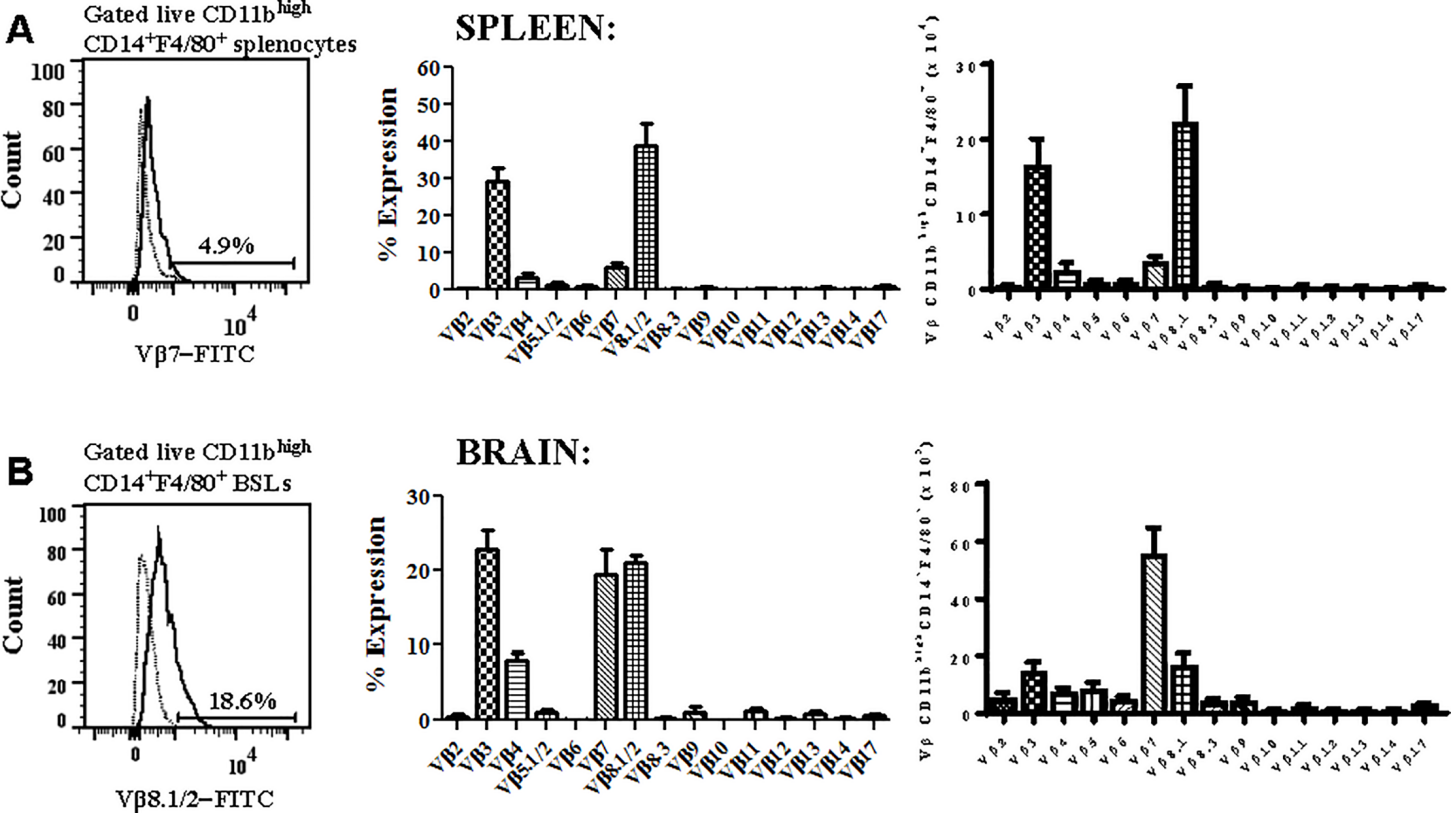
Brain sequestered macrophages preferentially express Vβ3, Vβ7, and Vβ8.1/2 during experimental cerebral malaria. *A*: C57BL/6 mice were infected with 10^6^ *Plasmodium berghei* ANKA parasites and T cell receptor Vβ usage was evaluated in splenic CD11b^high^CD14^+^F4/80^+^ macrophages of moribund mice (n = 5) on day 6 post-infection using a flow cytometry based mouse Vβ TCR Screening Panel. *B*: The type of and preference for Vβ TCR was also assessed in brain sequestered CD11b^high^CD14^+^F4/80^+^ macrophages during the cerebral phase of experimental cerebral malaria. Results shown depict the proportion of macrophages that express each Vβ chain and the total number of macrophages that express each Vβ chain after normalization for cell volume and are representative of three independent experiments.

We next measured the expression of 15 Vβ TCR subtypes on CD11b^high^CD14^+^F4/80^+^ macrophages in the spleen and brain on day 6 post-*Pb−A* infection by flow cytometry to assess the diversity of and preference for Vβ subtypes. In the spleen, CD11b^high^CD14^+^F4/80^+^ macrophages predominantly expressed the Vβ3 (29.3 ± 3.5%) or Vβ8.1/2 (39.0 ± 5.9%) TCR chain. In addition, 3.5 ± 1.2% and 6.1 ± 1.2% of splenic macrophages expressed the Vβ4 or Vβ7 TCR chain (Fig 8*A*). In the perfused brain, sequestered CD11b^high^CD14^+^F4/80^+^ macrophages predominantly expressed Vβ3, Vβ7 or Vβ8.1/2 (Fig 8*B*). However, when normalized for cell volume, brain sequestered macrophages preferentially express Vβ7. Interestingly, although Vβ7 expressing CD11b^high^CD14^+^F4/80^+^ macrophages were relatively rare in the spleen, it is the major subset in the brain. This suggests that while the Vβ7 population does not expand rapidly in response to *Pb−A* parasites, it preferentially migrates to and sequesters in the brain during ECM. Importantly, these results differ from TCR Vβ repertoire analysis of CD3^+^ T cells in *Pb−A* infected B10.D2 mice [38]. In this study, analysis of CD3^+^ T lymphocytes in the peripheral blood, lymph nodes, and spleen on day 7 post-infection showed a significant increase in Vβ8.1/2 but not Vβ3 or Vβ7 CD3^+^ T cells. Together, these results indicate that the Vβ repertoire of macrophages is distinct from that of T cells during *Pb−A* infection.

### TCRβ expression by the macrophage correlates with parasite burden on day 3 post-Pb−A infection

**Fig 9:**
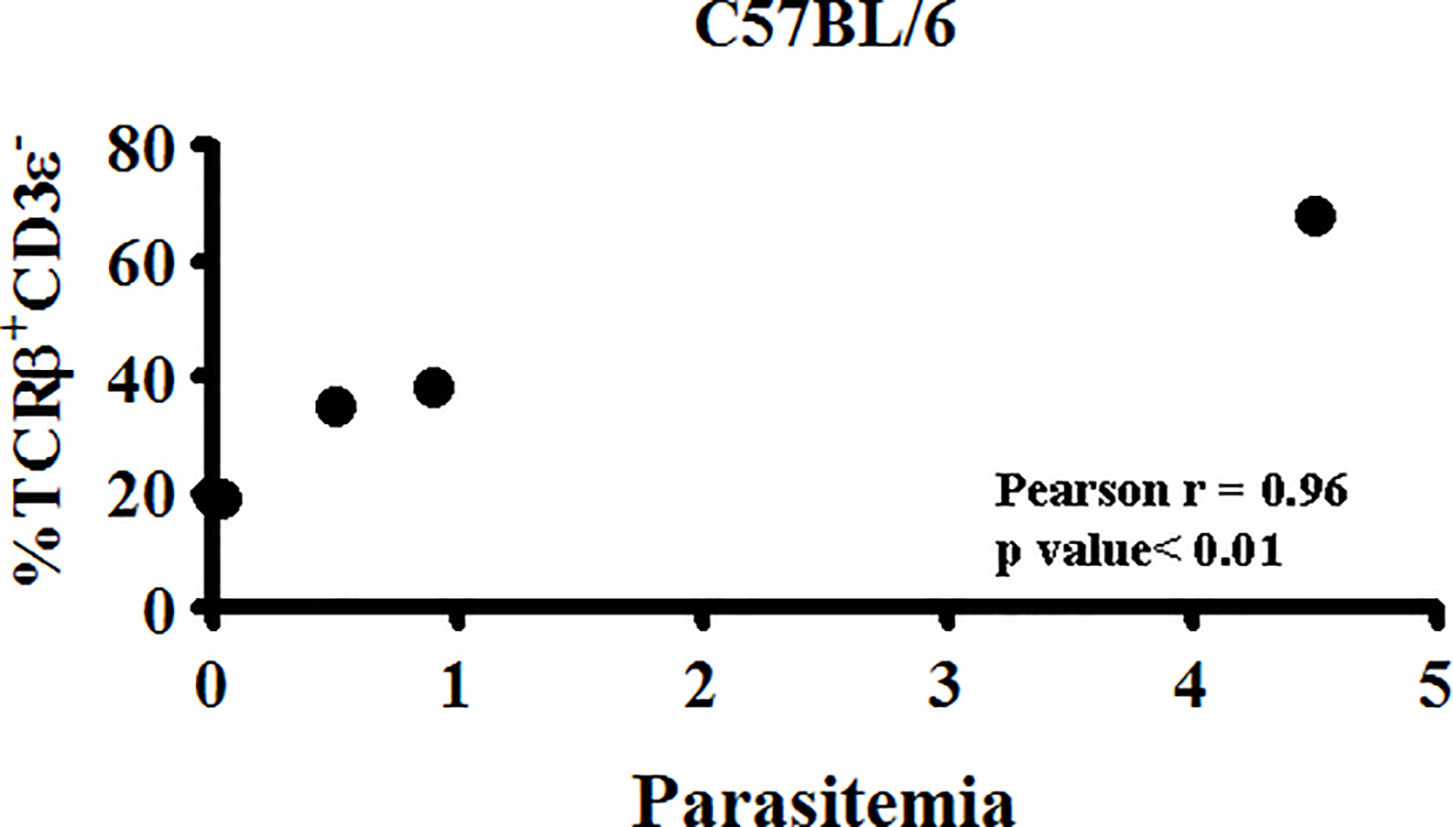
TCRβ expression by the macrophage correlates with *Plasmodium berghei* ANKA parasite burden. On day 3 post-infection, the correlation between the percentage of Ly6G^−^CD11b^high^CD14^+^F4/80^+^ macrophages that are TCRβ^+^CD3ε^−^ and peripheral parasitemia (parasitized erythrocytes/total erythrocytes x 100) in five individual mice was determined. Results shown are representative of four independent experiments. Pearson r = 0.96, P<0.01.

Remarkably, on day 3 post-infection, we noted a highly significant positive correlation (p≤0.01, R^2^ = 0.96, Pearson r correlation) between the proportion of CD11b^high^CD14^+^F4/80^+^ macrophages that express TCRβ and parasite burden in individual mice (Fig 9); a smaller percentage of macrophages were TCRβ^+^CD3ε^−^ in mice with low parasitemia compared to mice with high parasitemia. This striking correlation suggests that parasite burden may be driving the expansion of TCRβ−expressing macrophages and this novel population of TCRβ−expressing CD11b^high^CD14^+^F4/80^+^ macrophages may in turn play an important role in the regulation of parasite burden during a *Pb−A* infection.

### TCRβ expression correlates with enhanced phagocytosis of parasitized erythrocytes by macrophages

**Fig 10:**
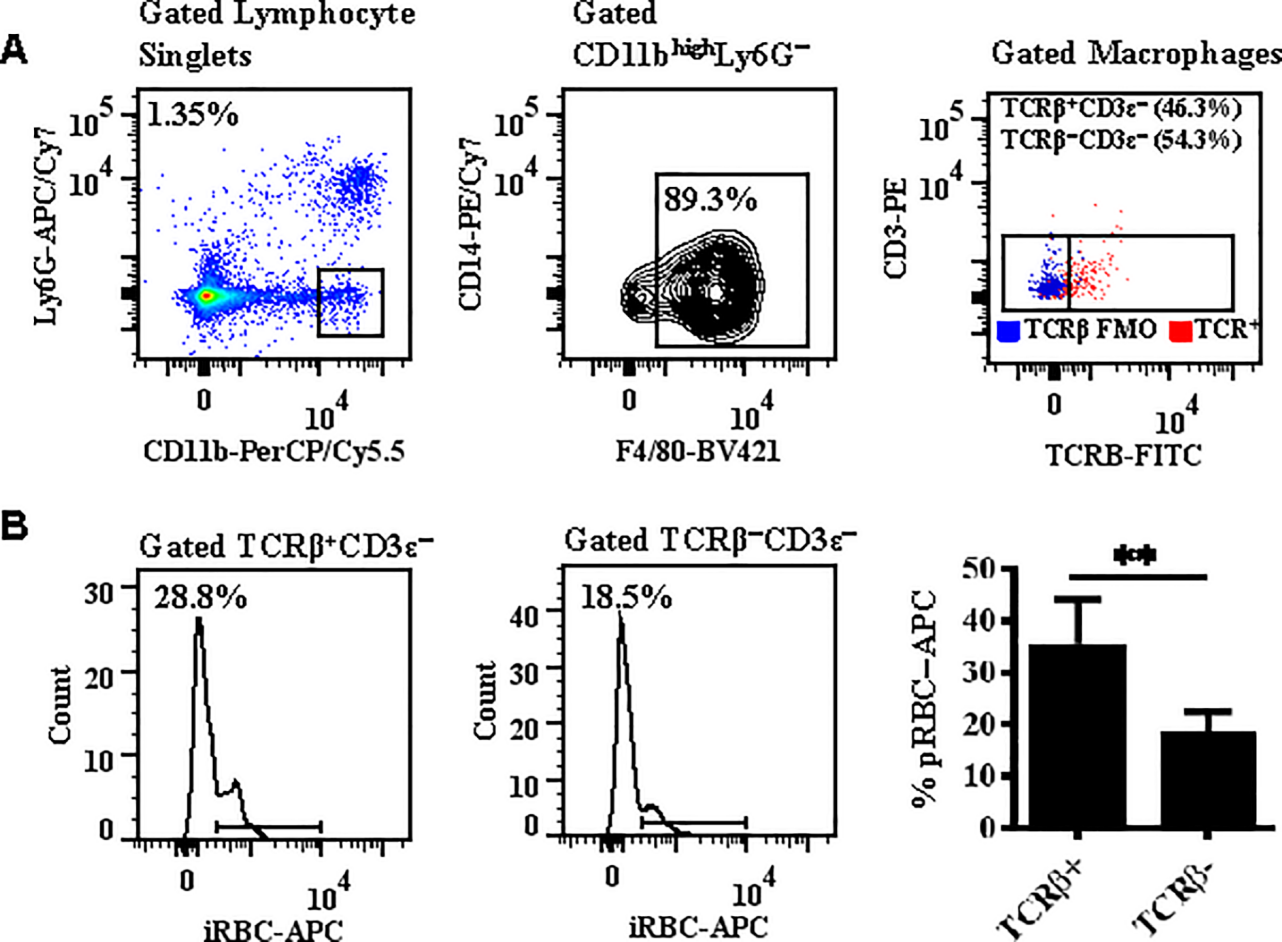
TCRβ expression correlates with enhanced phagocytosis of parasites by macrophages. *A*: Splenocytes and parasitized red blood cells (pRBCs) were isolated from C57BL/6 mice on day 3 post-infection with *Plasmodium berghei* ANKA. A 1:1 ratio of splenocytes (labeled with macrophage and T lymphocyte markers) and pRBCs (labeled with CellTrace^®^) were then incubated for 90 minutes to assess the effect of TCRβ expression by Ly6G^−^CD11b^high^F4/80^+^ macrophages on phagocytosis in an *in vitro* assay. *B*: Expression of TCRβ correlates with enhanced phagocytosis of pRBCs by macrophages. n = 3 for each phagocytosis assay. Results are presented as mean ± standard deviation and are a replicate of three independent experiments. Significant difference between TCRβ^+^ versus TCRβ^−^ macrophages is indicated as **P < 0.01. P−value was calculated using the Mann Whitney *U* test.

An important mechanism of immune regulation of parasite burden is phagocytosis of parasitized erythrocytes (pRBCs) by macrophages. To determine whether TCRβ expression influenced the phagocytic function of macrophages, splenocytes labeled with macrophage (CD11b, CD14, and F4/80) and T cell (TCRβ and CD3ε) markers and CellTrace™ labeled peripheral pRBCs isolated from mice on day 3 post-infection were incubated in a 1:1 ratio for 90 minutes and the ability of TCRβ^+^ versus TCRβ^−^ macrophages (Fig 10*A*) to phagocytose pRBCs was then compared by flow cytometry (Fig 10*B*). Remarkably, expression of TCRβ correlated with enhanced phagocytosis of pRBC by the macrophage; 35.4 ± 2.7% of TCRβ^+^ versus 18.6 ± 1.2% TCRβ^−^ macrophages were CellTrace™−APC positive by flow cytometric analysis (p<0.01, Mann Whitney *U* test) (Fig 10*B*).

### Transcriptional differences in TCRβ^+^ versus TCRβ^−^ macrophages

To better understand the molecular requirements for TCRβ expression and the immune effector mechanisms induced by TCRβ expression, we compared the transcriptome by microarray of TCRβ^+^ versus TCRβ^−^ Ly6G^−^CD11b^high^F4/80^+^CD3ε^−^CD4^−^CD8^−^ macrophages sorted from splenocytes of pooled mice on day 3 post-infection with *Pb−A*. Microarray was performed in quadruplicate. The input data was 28,603 genes. Using criteria of ± 3 fold change in expression and a *p* value of ≤ 0.01 (2-tailed Student *t* test), the output data was 73 protein coding genes. Next, we systematically categorized these genes into functional classes (S1 Table). Among the most highly differentially overexpressed genes are three subcomponents of the collagenous complement component C1q. The complement pathway is essential for the pathogenesis of ECM [39]. The C1q component has been shown to play a major role in stimulating phagocytosis by macrophages [40]; this is consistent with the enhanced phagocytic capacity that we observed in macrophages that express TCRβ.

Our microarray results also suggest that TCRβ−expressing macrophages express a unique signature of macrophage associated genes—Spic, Marco, Fcgr4, Cd300ld3, CD5l, and Timd4. Spic encodes a transcription factor that selectively controls the development of red pulp macrophages [41] which are thought to be involved in host defense against malaria [42]. Marco, a collagenous molecule with a distinct receptor domain (the SRCR domain), is upregulated by 9.7 fold in TCRβ^+^ versus TCRβ^−^ macrophages. This scavenger receptor-like molecule has been shown to be upregulated by proinflammatory stimuli in the nervous system [43]. Urokinase-type plasminogen activator, a peptidase with a trypsin-like domain, is upregulated by 4.1fold in TCRβ expressing macrophages. Urokinase-type plasminogen activator has been shown to be expressed at elevated levels in macrophages from arterial lesions in atherosclerosis [44] and lesion associated expression of its receptor (uPAR, CD87) has been documented in humans with CM [45]. Interestingly, we also observed the gene for the peptide messenger secretin (Sct) to be overexpressed by 3.9 fold in TCRβ−expressing macrophages. This points to an unusual signal released by these cells which is potentially significant given the presence of secretin receptors in the brain, the site of sequestration of these cells.

Lastly, CD8^+^ T lymphocyte associated genes (granzyme A, granzyme B, and perforin 1) and B lymphocyte associated genes (five immunoglobulin genes) were transcriptionally down-regulated by TCRβ−expressing macrophages. Although these results were unexpected, a previous study has shown that macrophages express granzyme B in human inflammatory diseases such as atherosclerosis and rheumatoid arthritis [46].

## Discussion

Monocytes/macrophages are equipped with a wide array of invariant receptors (e.g. Toll-like receptors, complement receptors, scavenger receptors, and Fc receptors) that facilitate the phagocytic clearance of invading pathogens and the removal of deformed, senescent, or dead host cells including red blood cells (RBCs) [47, 48]. Here we present the first report of the expression of combinatorial TCRβ immunoreceptor on macrophages in brain and spleen tissue during malaria infection. In *Pb−A* infected C57BL/6 mice, we found that 84.2 ± 2.3% of brain sequestered macrophages express TCRβ during the cerebral phase of ECM (Fig 2*A*). Additional experiments using a combination of AMNIS imaging (Fig 2*B*) and confocal microscopy (Fig 2*C*) confirm that TCRβ is uniformly expressed on the surface of brain sequestered macrophages. Through in depth phenotypic analysis, we also demonstrate that these TCRβ−expressing macrophages are CD3ε^−^CD4^−^CD8^−^ and Ly6G^−^ and require coexpression of CD14 and F4/80 for optimal expression of TCRβ on CD11b^high^ cells.

Here we report on a novel population of CD11b^high^CD14^+^F4/80^+^ macrophages that express combinatorial TCRβ during a *Pb−A* infection. This population of TCRβ−expressing macrophages is present at low levels in naïve mice, expands dramatically during *Pb−A* infection in both ECM susceptible C57BL/6 and resistant Balb/c mice, and migrates to and sequesters within the brain during the cerebral phase of ECM.

The TCRβ−expressing macrophages are Ly6G^−^ and CD3ε^−^CD4^−^CD8^−^, require coexpression of both CD14 and F4/80 on CD11b^high^ cells for optimal expression of TCRβ, and macrophage TCRβ undergoes productive gene rearrangements and displays preferential Vβ usage. Remarkably, on day 3 post-infection, there is a highly significant correlation between the proportion of macrophages that express TCRβ and parasite burden. Furthermore, we demonstrate that TCRβ expression also correlates with enhanced macrophage phagocytosis of parasitized erythrocytes. Lastly, we show through genome-wide transcriptional profiling of TCRβ^+^ versus TCRβ^−^ macrophages that TCRβ expression by the macrophage associates with a unique signature of macrophage associated genes, upregulation of the complement pathway, and down regulation of CD8^+^ T lymphocyte associated genes (granzyme A, granzyme B, and perforin 1) and B lymphocyte associated genes (five immunoglobulin genes).

In spite of numerous published reports, TCRβ expression by the macrophage may be considered unconventional. In carefully designed experiments, we verified that malaria-induced expression of TCRβ by the macrophage is not simply a consequence of 1) nonspecific binding of anti-TCRβ to an Fc receptor or cross-reactive epitope on the macrophage surface or 2) passive receptor expression via phagocytosis or trogocytosis (membrane swapping) of peripheral T cells. Measurement of TCRβ RNA in macrophages by PrimeFlow™, molecular analysis of the TCRβ repertoire by template switch PCR of macrophage RNA, and demonstration of preferential usage of Vβ by macrophages using a flow cytometry based Vβ TCR screening panel verify that TCR expression by the macrophage is not an artifact of nonspecific binding of anti-TCRβ to a non-TCRβ epitope or Fc receptor on the macrophage. In addition, abundant expression of both TCRβ transcript and protein in macrophages isolated from nude (which lack T cells) and *rag1* knockout (which lack T and B cells) mice eliminate the likelihood that peripheral T cells are the source of macrophage TCRβ. While absence of CD3ε on TCRβ−expressing macrophages and presence of abundant levels of both TCRβ transcript and protein on *rag1*^*tm1Mom*^ macrophages were unexpected results, these findings are supported by previous investigations of TCR expression by nonlymphoid cells [21] [24]. For example, Beham *et al* reported absence of CD3 on TCRαβ−expressing neutrophils and presence of TCRαβ on neutrophils isolated from V(D)J recombination-defective SCID mice and X-linked SCID humans. In addition, rare TCR recombination in *rag1* knockout mice despite the lack of functional T cells has previously been reported [49].

In summary, our studies have resulted in several important findings 1) during *Pb−A* infection, macrophages express TCRβ that undergoes productive gene rearrangements and displays preferred Vβ usage 2) TCRβ expression by the macrophage correlates with parasite burden and enhanced phagocytosis 3) the macrophage TCRβ-FITC MFI is significantly higher (1.7 fold) in C57BL/6 (ECM susceptible) versus Balb/c (ECM resistant) mice on day 4 post-infection and 4) microarray analysis indicates that TCRβ^+^ macrophages express a unique signature of macrophage associated genes, upregulate the complement system, and down regulate CD8^+^ T lymphocyte associated genes (granzyme A, granzyme B, and perforin 1) and B lymphocyte associated genes (five immunoglobulin genes). Our finding that TCRβ expression associates with an enhanced phagocytic capacity by the macrophage points towards a novel immunological adaption to control parasite growth during acute malaria when the adaptive immune response is still developing. Additionally, our results demonstrating that greater than 90% of brain sequestered macrophages express TCRβ during ECM suggest a role for TCRβ−expressing macrophages in disease pathogenesis.

Despite these novel findings, the immunological significance of TCRβ expression by macrophages during malaria is not clear. Several questions including the malaria antigen repertoire recognized by macrophage TCRβ, the parasite and host requirements for expansion and differentiation of TCRβ−expressing macrophages, the role of this novel population in the pathogenesis of ECM and SMA, and the effect of TCRβ on the biology and function of the macrophage deserve investigation in future studies.

## Supporting information

S1 Fig Gating strategy used to measure TCRβ expression on CD11b^high^CD14^+^F4/80^+^ macrophages in perfused brain tissue of moribund C57BL/6 mice. (TIF)

S2 Fig Gating strategy used to measure TCRβ expression on CD11b^high^CD14^+^F4/80^+^ macrophages in spleen tissue of C57BL/6 and Balb/c mice. (TIF)

S3 Fig The absolute number of CD11b^high^ cellular subsets in the spleen that are TCRβ^+^CD3ε^−^. (TIF)

S1 Table Transcriptome of TCRβ^+^ versus TCRβ^−^ macrophages. (DOCX)
