# Supplementary figures and images for: TCRβ-expressing macrophages induced by a pathogenic murine malaria correlate with parasite burden and enhanced phagocytic activity
Source: PLoS One. 2018 Jul 25;13(7):e0201043. doi: 10.1371/journal.pone.0201043 (PMC6059462; doi:10.1371/journal.pone.0201043)

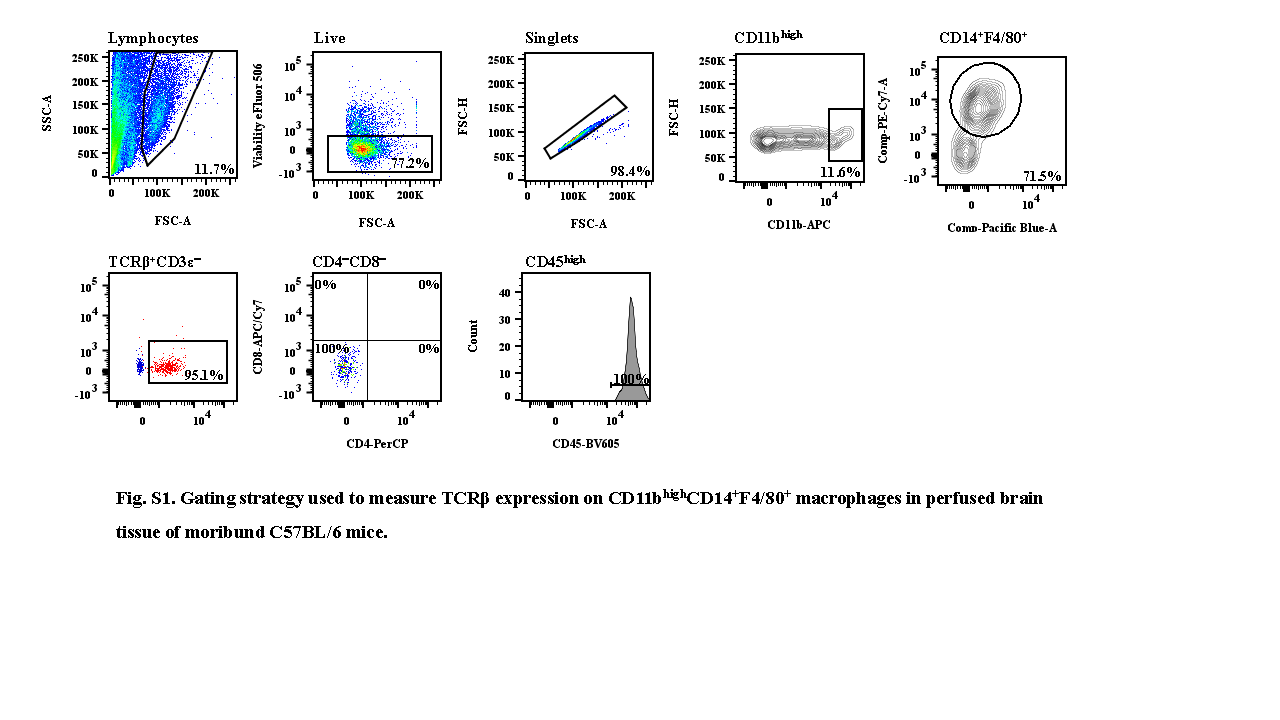

Supplement: S1 Fig — (TIF) [file pone.0201043.s001.tif]

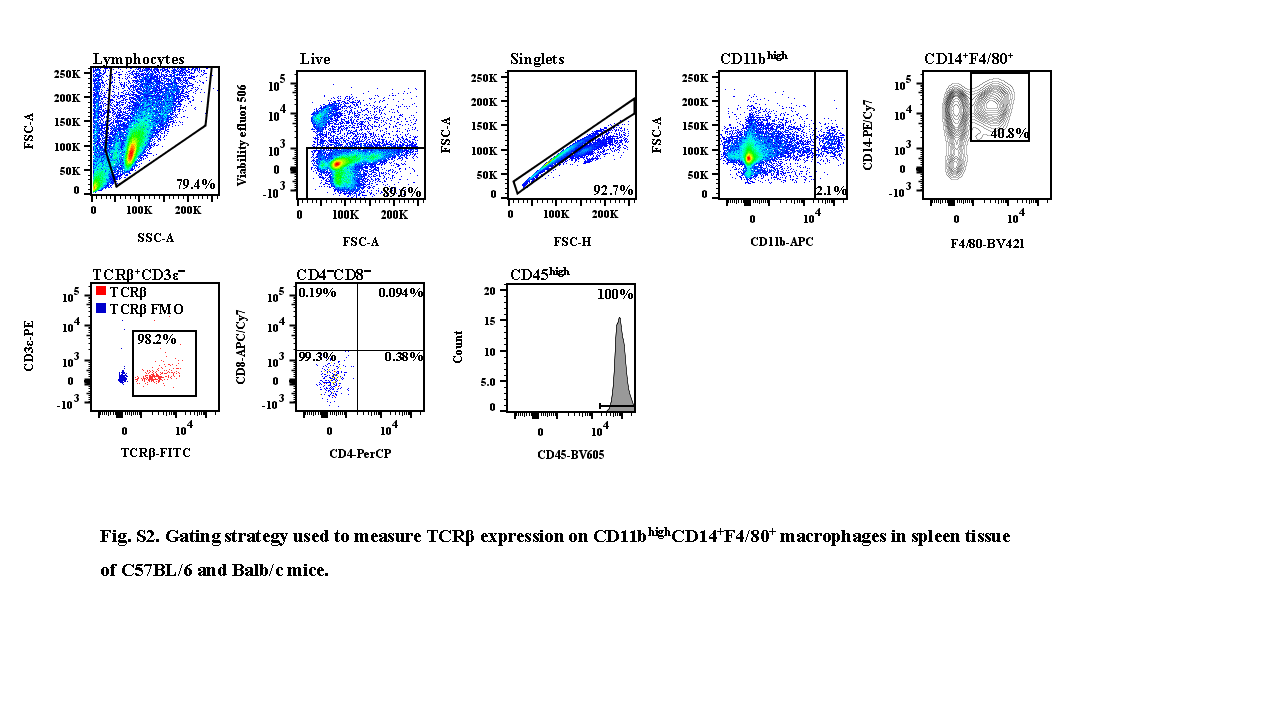

Supplement: S2 Fig — (TIF) [file pone.0201043.s002.tif]
