# Supplementary material for: TCRβ-expressing macrophages induced by a pathogenic murine malaria correlate with parasite burden and enhanced phagocytic activity
Source: PLoS One. 2018 Jul 25;13(7):e0201043. doi: 10.1371/journal.pone.0201043 (PMC6059462; doi:10.1371/journal.pone.0201043)
Supplement: S1 Table — (DOCX) [file pone.0201043.s004.docx]

Table S1. Transcriptome of TCRβ^+^ versus TCRβ^−^ macrophages.

| **Gene** | **Accession** | **Protein Domain Architecture** | **Average** | **mRNA Description** |
| --- | --- | --- | --- | --- |
| **Cell Adhesion and surface molecule** | | | | |
| Mmp13 | NP_032633.1 | SIG+PG_binding_1+Peptidase_M10+Hemopexin+Hemopexin+Hemopexin | 6.1972 | matrix metallopeptidase 13 (Mmp13), mRNA. |
| Pcolce2 | NP_083896.1 | SIG+CUB+CUB+OB_fold-Metalloproteinase(1uap_A) | 5.241 | procollagen C-endopeptidase enhancer 2 (Pcolce2), mRNA. |
| Adam19 | NP_001298138.1 | ADAM_CR+EGF+TM | 0.2516 | a disintegrin and metallopeptidase domain 19 (meltrin beta) (Adam19), transcript variant 1, mRNA. |
| Mmp8 | NP_032637.3 | SIG+PG_binding_1+Peptidase_M10+Hemopexin+Hemopexin+Hemopexin | 0.2274 | matrix metallopeptidase 8 (Mmp8), mRNA. |
| F5 | NP_032002.1 | SIG+Cu-oxidase+Cu-oxidase+Cu-oxidase+Cuoxidase+DISCOIDIN+DISCOIDIN | 0.1227 | coagulation factor V (F5), mRNA. |
| **Cytokine and First Messengers** | | | | |
| Igf1 | NP_034642.2 | SIG+Insulin | 8.7812 | insulin-like growth factor 1 (Igf1), transcript variant 1, mRNA. |
| Sct | NP_001296368.1 | SIG+Hormone_2 | 3.8729 | secretin (Sct), transcript variant 2, mRNA. |
| Ccl9 | P51670.1 | SH3(chemokine)(conservedCs) | 0.3235 | chemokine (C-C motif) ligand 9 (Ccl9), mRNA. |
| Xcl1 | NP_032536.1 | SIG+IL8 | 0.09 | chemokine (C motif) ligand 1 (Xcl1), mRNA. |
| Ifng | NP_032363.1 | IFN-gamma | 0.0754 | interferon gamma (Ifng), mRNA. |
| **Cytoskeleton** | | | | |
| Epb41l3 | Q9WV92.1 | FERM+4_1_CTD | 4.715 | erythrocyte membrane protein band 4.1 like 3 (Epb41l3), transcript variant X19, mRNA. |
| Dnah12 | Q3V0Q1.2 | EP1+DYN-MID-AAA | 3.5822 | dynein, axonemal, heavy chain 12 (Dnah12), transcript variant X1, mRNA. |
| Bbs9 | NP_851833.2 | BetaPropeller(PHTB1_N)+alpha-helical | 0.263 | Bardet-Biedl syndrome 9 (human) (Bbs9), transcript variant 1, mRNA. |
| Sep1 | P42209.2 | GTPase_Septin | 0.221 | septin 1 (Sept1), mRNA. |
| Rflnb | Q5SVD0.1 | refilinB | 0.195 | refilin B (Rflnb), mRNA. |
| Syne1 | NP_001334640.1 | CH+CH+Spectrin+Spectrin+Spectrin+Spectrin+KASH+TM | 0.0516 | spectrin repeat containing, nuclear envelope 1 (Syne1), transcript variant 4, mRNA. |
| **Immunity related effectors** | | | | |
| Rnase2a | NP_444343.2 | SIG+RnaseA | 0.2388 | ribonuclease, RNase A family, 2A (liver, eosinophil-derived neurotoxin) (Rnase2a), mRNA. |
| Mpo | P11247.2 | peroxidase | 0.2319 | myeloperoxidase (Mpo), mRNA. |
| Ncr1 | NP_034876.2 | SIG+IG+IG+TM | 0.2095 | natural cytotoxicity triggering receptor 1 (Ncr1), mRNA. |
| Pla2g3 | NP_766379.2 | SIG+Tox-PLA2 | 0.1527 | phospholipase A2, group III (Pla2g3), mRNA. |
| Gzmb | NP_038570.1 | SIG+TRYPSERINEPROTEASE | 0.1193 | granzyme B (Gzmb), mRNA. |
| Prf1 | NP_035203.3 | SIG+MACPERFORIN+C2 | 0.1076 | perforin 1 (pore forming protein) (Prf1), mRNA. |
| Gzma | P11032.2 | SIG+TRYPSERINEPROTEASE | 0.0717 | granzyme A (Gzma), mRNA. |
| **Immunity related Cell Adhesion and surface molecules** | | | | |
| Cd5l | Q9QWK4.3 | SIG+SRDOMAIN+SRDOMAIN+SRDOMAIN | 27.3925 | CD5 antigen-like (Cd5l), mRNA. |
| Fcna | O70165.1 | SIG+Collagen+Fibrinogen_C | 17.8472 | ficolin A (Fcna), mRNA. |
| Vcam1 | P29533.1 | SIG+IG+IG+IG+IG+IG+IG+IG+TM | 10.6666 | vascular cell adhesion molecule 1 (Vcam1), mRNA. |
| C1qa | P98086.2 | jelly roll | 10.3539 | complement component 1, q subcomponent, alpha polypeptide (C1qa), mRNA. |
| C1qb | P14106.2 | SIG+Collagen+C1q-jellyroll | 5.3732 | complement component 1, q subcomponent, beta polypeptide (C1qb), mRNA. |
| C1qc | Q02105.2 | jelly roll | 9.0866 | complement component 1, q subcomponent, C chain (C1qc), mRNA. |
| Marco | NP_034896.1 | TM+Collagen+Collagen+Collagen+Collagen+SRDOMAIN | 9.7395 | macrophage receptor with collagenous structure (Marco), mRNA. |
| Mertk | NP_032613.1 | SIG+IG+FN3+TM+CR-Kinase | 6.1501 | c-mer proto-oncogene tyrosine kinase (Mertk), mRNA. |
| Tspan4 | Q9DCK3.1 | SIG+TM+TM+helicalbundle+TM | 5.8896 | tetraspanin 4 (Tspan4), transcript variant 1, mRNA. |
| Timd4 | NP_848874.3 | SIG+IG+TM | 5.6666 | T cell immunoglobulin and mucin domain containing 4 (Timd4), mRNA. |
| Cd300ld3 | Q6SJQ5.1 | SIG+IG+TM | 4.5667 | CD300 molecule like family member D3 (Cd300ld3), mRNA. |
| Plau | NP_032899.1 | SIG+TRYPSERINEPROTEASE | 4.133 | plasminogen activator, urokinase (Plau), mRNA. |
| Lilra5 | XP_006539826.1 | IG+IG+TM | 3.7578 | leukocyte immunoglobulin-like receptor, subfamily A (with TM domain), member 5 (Lilra5), mRNA. |
| Fcgr4 | A0A0B4J1G0.1 | SIG+IG+IG+IG+IG+IG+TM | 3.6393 | Fc receptor, IgG, low affinity IV (Fcgr4), mRNA. |
| Lilra6 | NP_035220.2 | SIG+IG+IG+IG | 0.2807 | leukocyte immunoglobulin-like receptor, subfamily A (with TM domain), member 6 (Lilra6), transcript variant 1, mRNA. |
| Itga1 | NP_001028400.2 | SIG+VWA+IG+TM | 0.2537 | integrin alpha 1 (Itga1), mRNA. |
| Igk | CAI54295.1 | IG | 0.2191 | Immunoglobulin kappa chain complex |
| Ighm | P01872.2 | IG+IG+IG+IG | 0.1952 | Immunoglobulin heavy constant mu |
| Ngp | NP_032720.2 | SIG+Cystatin+Cystatin | 0.195 | neutrophilic granule protein (Ngp), mRNA. |
| Igj | P01592.4 | SIG+IG | 0.194 | Immunoglobulin joining chain |
| Nkg7 | NP_077215.2 | SIG+TM+TM+TM | 0.1853 | natural killer cell group 7 sequence (Nkg7), mRNA. |
| Klre1 | EDK99935.1 | SIG+CLECTIN | 0.1679 | killer cell lectin-like receptor family E member 1 (Klre1), mRNA. |
| Igkv4-72 | AAF69328.1 | IG | 0.1602 | Immunoglobulin kappa chain variable 4-72 |
| Klra9 | NP_034781.2 | TM+CLECTIN | 0.144 | killer cell lectin-like receptor subfamily A, member 9 (Klra9), mRNA. |
| Camp | P51437.2 | SIG+Cystatin+helical(2K60A) | 0.0932 | cathelicidin antimicrobial peptide (Camp), mRNA. |
| Prg2 | NP_032946.1 | SIG+CLECTIN | 0.083 | proteoglycan 2, bone marrow (Prg2), mRNA. |
| Iglv1 | P01727.1 | SIG+IG | 0.0749 | Immunoglobulin lambda variable 1 |
| Il1rl1 | P14719.2 | SIG+IG+IG+TM+TIR | 0.0646 | interleukin 1 receptor-like 1 (Il1rl1), transcript variant 1, mRNA. |
| Retnlg | Q8K426.1 | SIG+Resistin | 0.0278 | resistin like gamma (Retnlg), mRNA. |
| **Metabolism** | | | | |
| Akr1b7 | P21300.4 | TIMbarrel | 5.6627 | aldo-keto reductase family 1, member B7 (Akr1b7), mRNA. |
| Cmbl | Q8R1G2.1 | AB Hydrolase | 4.971 | carboxymethylenebutenolidase-like (Pseudomonas) (Cmbl), mRNA. |
| Gatm | NP_080237.1 | Amidinotransferase | 4.6577 | glycine amidinotransferase (L-arginine glycine amidinotransferase) (Gatm), mRNA. |
| Fads1 | Q920L1.1 | Cytochrome+TM+TM+TM+TM | 3.4718 | fatty acid desaturase 1 (Fads1), mRNA. |
| **Signaling** | | | | |
| Adrb1 | P34971.2 | 7TMeuk | 5.1414 | adrenergic receptor, beta 1 (Adrb1), mRNA. |
| Hebp1 | NP_038574.3 | SHS2 | 4.0607 | heme binding protein 1 (Hebp1), mRNA. |
| Padi4 | Q9Z183.3 | Cupredoxin+IG+Pentein | 0.2814 | peptidyl arginine deiminase, type IV (Padi4), mRNA. |
| Arap3 | Q8R5G7.3 | SAM+PH+FERM+PH | 0.1822 | ArfGAP with RhoGAP domain, ankyrin repeat and PH domain 3 (Arap3), transcript variant 1, mRNA. |
| Rgcc | NP_079703.2 | RGCC(Pfam) | 0.1681 | regulator of cell cycle (Rgcc), mRNA. |
| Map3k6 | NP_057902.5 | TPR_repeats+STKinase | 0.148 | mitogen-activated protein kinase kinase kinase 6 (Map3k6), mRNA. |
| Rab44 | Q8CB87.1 | sGTP | 0.1109 | RAB44, member RAS oncogene family (Rab44), mRNA. |
| Tesc | NP_067319.2 | EF-HAND | 0.1082 | tescalcin (Tesc), mRNA. |
| **Transcription** | | | | |
| Spic | NP_035591.3 | ETS | 3.1395 | Spi-C transcription factor (Spi-1/PU.1 related) (Spic), mRNA. |
| Eaf2 | Q91ZD6.1 | EAF (RNA polI subunit - tripleBarrel) | 0.1655 | ELL associated factor 2 (Eaf2), transcript variant 1, mRNA. |
| Cebpe | NP_997014.1 | BZIP | 0.1203 | CCAAT/enhancer binding protein (C/EBP), epsilon (Cebpe), mRNA. |
| **Transporter** | | | | |
| Slc40a1 | Q9JHI9.1 | TM+TM+TM+TM+X-alphahelical+TM+TM+TM+TM+TM+TM | 12.7266 | solute carrier family 40 (iron-regulated transporter), member 1 (Slc40a1), mRNA. |
| Abcc3 | NP_083876.3 | TM+TM+TM+TM+TM+TM+TM+TM+TM+TM+ABC-ATPase+TM+TM+TM+TM+ABC-ATPase | 6.2601 | ATP-binding cassette, sub-family C (CFTR/MRP), member 3 (Abcc3), mRNA. |
| Clic5 | Q8BXK9.1 | Thioredoxin-like+alpha-helical | 4.0562 | chloride intracellular channel 5 (Clic5), mRNA. |
| Slc45a3 | NP_666089.1 | SIG+TM+TM+TM+TM+TM+TM+TM+TM+TM+X3+TM | 3.923 | solute carrier family 45, member 3 (Slc45a3), transcript variant 1, mRNA. |
| **Miscellaneous** | | | | |
| Hbb-bt | BAB27380.1 | Globin | 5.65 | hemoglobin, beta adult t chain (Hbb-bt), mRNA. |
| Hba-a1 | NP_032244.2 | Globin | 4.6895 | hemoglobin alpha, adult chain 1 (Hba-a1), mRNA. |
